# Chromosome-scale genome assembly of Australian finger lime and resequencing reveal the hidden diversity of Oceanian citrus

**DOI:** 10.1093/hr/uhag213

**Published:** 2026-06-02

**Authors:** Barbara Hufnagel, Monica Alves, Quentin Piet, Alexis Dereeper, Delphine Giraud, Leny Calvez, Caroline Belser, Karine Labadie, Simone Duprat, Corinne Cruaud, Benjamin Istace, Franck Curk, Fredson Dos Santos Menezes, Khadidiatou Diop, Maëva Miranda, Yann Froelicher, Malcolm Smith, Pablo Aleza, Stéphane Lebegin, Carole Martin, Fabienne Micheli, Pierre Mournet, Gaetan Droc, Leandro Peña, Nelson Wulff, Raphaël Morillon, Patrick Wincker, Jean-Marc Aury, Arnaud Lemainque, Patrick Ollitrault

**Affiliations:** CIRAD, UMR AGAP Institute, Petit-Bourg F-97170, France; UMR AGAP Institute, Univ Montpellier, CIRAD, INRAE, Institut Agro, F-34090 Montpellier, France; Fundo de Defesa da Citricultura, 14807-040, Araraquara, Brazil; CIRAD, UMR AGAP Institute, Petit-Bourg F-97170, France; UMR AGAP Institute, Univ Montpellier, CIRAD, INRAE, Institut Agro, F-34090 Montpellier, France; UMR PHIM, IRD, F-34394 Montpellier, France; UMR AGAP, INRAE, Institut Agro Montpellier, CIRAD, University of Montpellier, F-20230 San Giuliano, France; Fundo de Defesa da Citricultura, 14807-040, Araraquara, Brazil; Génomique Métabolique, Genoscope, Institut François Jacob, CEA, CNRS, Univ Evry, Université Paris-Saclay, Evry F-91057, France; Genoscope, Institut François Jacob, CEA, CNRS, Univ Evry, Université Paris-Saclay, Evry F-91057, France; Génomique Métabolique, Genoscope, Institut François Jacob, CEA, CNRS, Univ Evry, Université Paris-Saclay, Evry F-91057, France; Genoscope, Institut François Jacob, CEA, CNRS, Univ Evry, Université Paris-Saclay, Evry F-91057, France; Génomique Métabolique, Genoscope, Institut François Jacob, CEA, CNRS, Univ Evry, Université Paris-Saclay, Evry F-91057, France; UMR AGAP, INRAE, Institut Agro Montpellier, CIRAD, University of Montpellier, F-34060 Montpellier, France; Centro de Biotecnologia e Genética, Departamento de Ciências Biológicas, Universidade Estadual de Santa Cruz, Ilhéus 45662-900, Brazil; CNRS/UPVD, Plant Genome and Development (LGDP), Perpignan F-66100, France; UMR AGAP Institute, Univ Montpellier, CIRAD, INRAE, Institut Agro, F-34090 Montpellier, France; CIRAD, UMR AGAP Institut, F-34398 Montpellier, France; UMR AGAP Institute, Univ Montpellier, CIRAD, INRAE, Institut Agro, F-34090 Montpellier, France; CIRAD, UMR AGAP Institut, F-20230 San Giuliano, France; Department of Agriculture and Fisheries, Bundaberg Research Station, Qld 4670 Bundaberg, Australia; Departamento de Citricultura y Producción Vegetal, Instituto Valenciano de Investigaciones Agrarias (IVIA), 46113 Moncada, Spain; Institut Agronomique Néo-Calédonien, Station de Recherche Agronomique de Pocquereux, 98880 La Foa, Nouvelle Calédonie; Institut Agronomique Néo-Calédonien, Station de Recherche Agronomique de Pocquereux, 98880 La Foa, Nouvelle Calédonie; UMR AGAP Institute, Univ Montpellier, CIRAD, INRAE, Institut Agro, F-34090 Montpellier, France; CIRAD, UMR AGAP Institut, F-34398 Montpellier, France; UMR AGAP Institute, Univ Montpellier, CIRAD, INRAE, Institut Agro, F-34090 Montpellier, France; CIRAD, UMR AGAP Institut, F-34398 Montpellier, France; UMR AGAP Institute, Univ Montpellier, CIRAD, INRAE, Institut Agro, F-34090 Montpellier, France; CIRAD, UMR AGAP Institut, F-34398 Montpellier, France; Instituto de Biología Molecular y Celular de Plantas, Consejo Superior de Investigaciones Científicas, Universidad Politécnica de 46022 Valencia, Valencia, Spain; Fundo de Defesa da Citricultura, 14807-040, Araraquara, Brazil; UMR AGAP Institute, Univ Montpellier, CIRAD, INRAE, Institut Agro, F-34090 Montpellier, France; CIRAD, UMR AGAP Institut, F-34398 Montpellier, France; Génomique Métabolique, Genoscope, Institut François Jacob, CEA, CNRS, Univ Evry, Université Paris-Saclay, Evry F-91057, France; Génomique Métabolique, Genoscope, Institut François Jacob, CEA, CNRS, Univ Evry, Université Paris-Saclay, Evry F-91057, France; Genoscope, Institut François Jacob, CEA, CNRS, Univ Evry, Université Paris-Saclay, Evry F-91057, France; UMR AGAP Institute, Univ Montpellier, CIRAD, INRAE, Institut Agro, F-34090 Montpellier, France; Fundo de Defesa da Citricultura, 14807-040, Araraquara, Brazil; CIRAD, UMR AGAP Institut, F-34398 Montpellier, France

## Abstract

Oceanian citrus species, including wild taxa native to Australia and Papua New Guinea, form a genetically distinct clade within the *Citrus* L. (1753) genus. These species remain largely underexplored, despite their adaptation to diverse environments and relevance for citrus improvement. To support their use in breeding and evolutionary studies, we generated a high-quality, chromosome-scale genome assembly of an Australian finger lime accession (SRA 1002), a natural interspecific hybrid. The genome was assembled using long-read sequencing, optical mapping, and a high-density genetic map, resulting in nine pseudomolecules covering over 97% of the genome. Using this reference, we analyzed whole-genome resequencing data from 132 accessions representing the diversity of Asian and Oceanian citrus. Variant calling across the dataset produced a high-resolution catalogue of single nucleotide polymorphisms (SNPs) and derived database of SNPs fully discriminant of 20 *Citrus* species (diagnostic SNPs, DSNPs), enabling detailed exploration of inter- and intraspecific diversity. The data reveal a strong population structure within the group with clear heterozygosity variation between ancestral species and admixed accessions, reflecting their complex evolutionary history and hybridization patterns. This study provides the first integrated genomic framework for Oceanian citrus diversity, offering essential tools for downstream applications in citrus breeding, conservation, and evolutionary genomics. The resources generated lay the groundwork for future association studies and the targeted introgression of beneficial traits into cultivated citrus.

## Introduction

Citrus is among the most important fruit crops worldwide, cultivated in more than 140 countries for its economic, nutritional, and cultural significance. The genus *Citrus*, as currently circumscribed [1, 2], includes the cultivated gene pool resulting from a reticulate lineage of interrelated species that originated from a small number of ancestral taxa in Southeast Asia [2]. Extensive interspecific hybridization, coupled with long-standing clonal propagation and apomixis, has fixed elite agronomic traits in cultivated varieties but has also drastically eroded genetic diversity within modern breeding germplasm [3–5]. This genetic narrowing limits the potential for developing resilient citrus cultivars, especially under emerging threats such as Huanglongbing (HLB), a devastating bacterial disease that threatens citrus production worldwide [6].

In contrast to the cultivated citrus gene pool, the Oceanian citrus species, comprising several taxa formerly classified under *Microcitrus Swin*gle (1915)*, Eremocitrus* Swingle (1914), *Clymenia* Swingle *(1939)*, and *Oxanthera* Montrouz (1860) [7], represent an underexplored reservoir of genetic and phenotypic diversity. These species, now included in the *Citrus* genus [1, 2], native to Australia, Papua New Guinea, and New Caledonia, occupy diverse ecological niches ranging from the arid interior (*Citrus glauca* [Lindl.] Burkill [1932]) to the tropical rainforests of Queensland (*Citrus australasica* F. Muell., *Citrus australis* (Mudie) Planch (1858), *Citrus inodora* F.M. Bailey (1889)) [8]. They form a monophyletic group distinct from the Asian citrus lineages, as demonstrated by nuclear phylogenomic analyses [9]. Oceanian citrus species exhibit valuable agronomic traits, including drought and cold tolerance, short juvenility, and, most notably, resistance or tolerance to HLB or greening [10, 11], caused by the phloem-limited bacterium *Candidatus Liberibacter asiaticus* (*Clas*). HLB is currently the most destructive disease affecting citrus worldwide, causing rapid tree decline and reduced yields. In Florida, its impact has been devastating, with citrus production dropping by roughly 70%–80%, severely damaging the industry.

Overall, HLB is reshaping the global citrus industry, widening regional disparities and posing a long-term threat to citrus production.

Recent studies have underscored the promise of these species in citrus improvement programs. Hybrids between Oceanian and Asian citrus have already been developed and evaluated, revealing the potential to introgress resistance traits into elite germplasm [12] and to develop new rootstocks [13]. Nevertheless, a comprehensive understanding of the genomic architecture and evolutionary dynamics of these wild species has been limited by the lack of high-quality, chromosome-scale reference genomes. Only in the past 2 years have phased genome assemblies been reported for several Australian taxa, including *C. australasica*, *C. australis*, *C. glauca*, and *C. inodora*, revealing structural variations, unique gene families, and lineage-specific expansions related to pathogen defense and stress adaptation [14, 15].

Among these species, *C. australasica* (Australian finger lime, AFL) has gained particular attention for its horticultural and culinary value, as well as its broad phenotypic variability in fruit morphology, color, and flavor [16]. Its ease of hybridization with other citrus species has made it a key parent in breeding programs aimed at improving fruit quality and disease resistance. However, preliminary GBS studies (our unpublished data under the PreHLB H2020 project) have shown that several AFL accessions maintained in French, Spanish, and Brazilian germplasm banks were introgressed with *C. australis*, forming complex interspecific mosaics. Understanding the genomic consequences of such introgression events and the distribution of structural variants across Oceanian lineages remains an open question.

Here, we present a chromosome-scale, high-contiguity genome assembly of an AFL accession SRA 1002 (AFL SRA 1002), a suspected interspecific backcross hybrid *C. australasica* × (*C. australis* × *C. australasica*), maintained at the Citrus Biological Resource Center (INRAE-CIRAD) in Corsica [17]. This finger lime has been cultivated for several years in Guadeloupe (French West Indies) under high HLB pressure and has shown neither symptom nor *Clas* multiplication. Its apparent resistance to HLB was a major motivation for *de novo* assembly. Using a combination of long-read Nanopore sequencing, PCR-free Illumina reads, Bionano optical maps, and a high-density interspecific genetic map, we anchored over 97% of the genome into nine pseudomolecules. We further analyzed whole-genome resequencing data of 132 accessions (including 54 new resequencing) representing the diversity of Asian and Oceanian *Citrus* to characterize population structure, detect interspecific introgressions, and identify diagnostic single nucleotide polymorphisms (DSNPs) across clades.

This work provides the first integrated genomic framework for the study of Oceanian *Citrus* diversity. It establishes foundational resources for evolutionary analyses, comparative genomics, and breeding applications aimed at exploiting wild diversity for improving stress tolerance and disease resistance in cultivated citrus.

## Results

### Australian finger lime sequencing and assembly

The reference genome of AFL SRA 1002 was assembled using a hybrid strategy that combined Oxford Nanopore long reads, PCR-free Illumina short reads, and Bionano optical maps to maximize contiguity and accuracy in a highly heterozygous, repeat-rich citrus genome. Nanopore reads enabled long-range contig assembly, Illumina reads ensured base-level polishing, and Bionano optical maps provided orthogonal long-range validation to detect/correct misassemblies and support scaffold ordering and orientation during pseudochromosome construction. The efficiency of this approach was first demonstrated with banana genomes by Belser *et al.* [18]. The initial assembly comprised 403 scaffolds totaling 450.8 Mb, with high contiguity (N50 = 28.4 Mb; N80 = 5.4 Mb) (Table S1; Supplementary Note 1). K-mer analysis using GenomeScope 2.0 estimated a genome size of ~338 Mb and a high heterozygosity rate, consistent with the hybrid origin of this accession (Fig. S1A).

The admixture analysis based on DSNPs (see ‘Diagnostic SNPs reveal clade differentiation and hybrid ancestry’) from WGS data of 132 accessions confirmed that AFL SRA 1002 is not a pure *C. australasica* but an interspecific hybrid, showing alternating homozygous *C. australasica* and heterozygous *C. australis*/*C. australasica* regions (Fig. 1A). These findings support a backcross origin: *C. australasica* × (*C. australasica* × *C. australis*), consistent with the presence of *C. australasica* cytoplasm.

**Figure 1:**
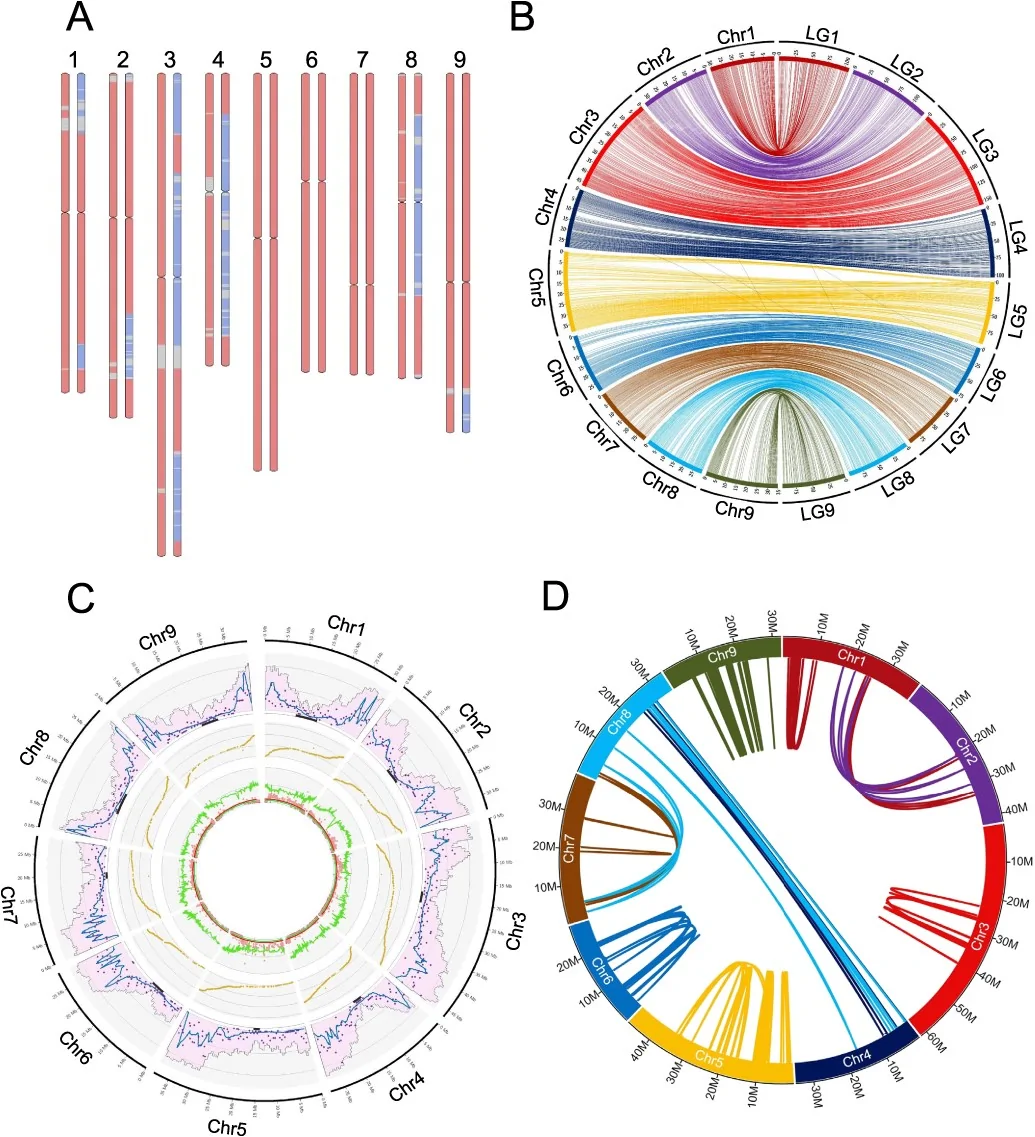
Overview of genome structure and quality metrics in AFL SRA 1002. (A) Mosaic structure of AFL SRA 1002, which revealed a backcross origin: *C. australasica ×* (*C. australasica × C. australis*). Genomic segments assigned to *C. australasica*, *C. australis*, and regions of undetermined origin are distinguished by different colors: pink, light blue, and grey, respectively. (B) Anchoring of the AFL SRA 1002 assembly to the *C. australis* × *C. inodora* genetic map. Each ribbon connects a genomic position from the pseudochromosome assembly (Chr) to its corresponding position in one of the nine linkage groups (LG). The strong one-to-one correspondence and limited cross-linking support the structural accuracy of the assembly. (C) Synthesis of parameter distribution along the genome. External layers: gene density (pink histogram), marker density (purple dots), and recombination landscape (blue line). Inner layers: Marey map (*y*-axis: genetic position) and segregation distortion analysis. Green line: *C. australis* haplotype contribution; brown dots: chi-square test *q*-values (based on expected 0.5/0.5 segregation); red line: significance threshold at *q* = 0.05. (D) Intragenomic segmental duplications. Circular representation of syntenic blocks > 7.6 kb identified by SynMap. Lines connect homologous segments between and within chromosomes.

Dotplot comparisons against the *Citrus reticulata* CITRE pseudochromosome assembly [19] revealed scaffold duplications, particularly in heterozygous interspecific regions (Fig. S1B). To correct for these redundancies, a curated assembly was generated by removing the duplicated scaffolds and reordering the remaining ones based on synteny and collinearity with the *C. reticulata* genome, in line with the conserved macrosynteny across Asian and Oceanian citrus species [20].

The resulting pseudomolecule assembly comprised 13 scaffolds spanning 300.85 Mb, representing 96.86% of the total genome length. These were anchored into nine pseudomolecules. Unanchored scaffolds were concatenated with 100 ‘N’ spacers to form a single unplaced scaffold (‘Chr Un’), totaling 9.77 Mb. A first genetic map of a *C. australis × C. inodora* hybrid was produced using the *Citrus clementina* V1.0 reference genome from the GBS data of a progeny of 171 ‘Fortune’ mandarin (*C. reticulata*) × (*C. australis × C. inodora*) hybrids [20]. We performed a new variant calling and genetic mapping from the same data using the new AFL assembly in pseudochromosome as template. A total of 2622 diallelic SNP markers were successfully mapped in nine linkage groups encompassing 943.61 cM (Table S2).

The genome assembly and genetic map were highly syntenic and collinear (Fig. 1B), with only nine markers (0.34%) discordant for synteny and a Spearman collinearity coefficient of 0.9997 ± 0.0001. These results validate the structural quality of the AFL genome assembly. The recombination landscape study revealed large genomic regions with very low recombination levels corresponding to low-gene-density areas (Fig. 1C). The concordance between these two parameters was used to estimate the location of the centromeres (black lines on the basis of external figures of Fig. 1C). Genomic regions with significant segregation distortion were observed in Chr 1, Chr 3, and Chr 5. In all cases, they corresponded to areas with a deficit in the transmission of the *C. australis* haplotype.

The global synteny and collinearity of the AFL assembly and other *Citrus* species were checked by the anchoring of the consensus genetic map of the *Citrus* genus that includes 10 756 markers [20]. It resulted in evidence for high synteny (96.72%) and collinearity (Spearman coefficient = 0.9974 ± 0.0018; Fig. S1C).

### Genome assembly statistics and gene annotation

The final AFL genome assembly comprised nine pseudochromosomes totaling 300.8 Mb and an additional 9.7 Mb of unanchored scaffolds. Transcriptomic data from flowers, leaves, and fruit pulp (196.4 Gb total) were used for gene annotation. BUSCO completeness exceeded 91% in most tissues, except fruit pulp (76%) (Table S3). Using the EuGene pipeline, 28 266 protein-coding genes were predicted (Table 1), distributed according to recombination-rich regions, consistent with other citrus genomes.

**Table 1:** Size and number of genes per pseudochromosome of the AFL genome.

**Pseudo-Chr**	**Size (bp)**	**Gene number**
1	30 981 035	2830
2	33 420 155	3459
3	46 787 044	4818
4	28 396 107	2781
5	38 550 532	3573
6	29 000 654	2636
7	29 262 762	2832
8	29 613 850	2537
9	34 833 955	2775
Un	9 772 391	25
Total	300 846 094	28 266

### Intragenomic segmental duplications in the AFL genome

Analysis of intragenomic duplications in the AFL SRA 1002 genome, using SynMap [21], with a minimum of five collinear genes per block, identified 1379 duplicated genes grouped into 109 syntenic blocks. These blocks ranged in size from 37.8 kb to 1.69 Mb, with a mean length of 174.2 kb and a median of 124.5 kb. For visualization, only 131 blocks exceeding 7.6 kb were retained, corresponding to the upper 5% of duplication sizes (Fig. 1D).

The resulting duplication landscape highlights a clear predominance of intrachromosomal duplications, particularly on Chr 9, which harbors the highest number of duplicated genes, and Chr 5, which engages extensively in interchromosomal exchanges. Notably, a strong syntenic connection is observed between Chr 4 and Chr 8, suggesting potential ancient segmental translocations or duplications mediated by transposable elements (TEs). Beyond these structural patterns, a subset of duplicated genes encodes ABC transporters, oxidoreductases, and putative transcriptional regulators, reflecting functional diversification. Together, these findings support the role of segmental duplications in shaping the dynamic architecture of the AFL genome and in providing evolutionary raw material for subfunctionalization, neofunctionalization, or pseudogenization.

### Conservation of citrus repeat diversity in AFL SRA 1002, but also minor novelties

Annotation of repeated sequences revealed that AFL SRA 1002 chromosomes contained a large amount of TEs, representing 42.55% of the genome, with 31.09% of retrotransposons and 10.50% of DNA transposons (Fig. 2A; Table 2). Among TEs identified in *C. australasica*, 99% has already been characterized in other citrus species [22]. However, *de novo* annotation allowed us to assemble 25 new consensus sequences of TEs found exclusively in AFL SRA 1002, covering 0.17% of the genome with 1276 copies. These TEs are mainly LTR retrotransposons, LINEs, and MITEs (9, 6, and 5 consensus, respectively; Table S4). Interestingly, 363 and 449 truncated copies of two different LINEs were identified, suggesting ancient accumulation events in AFL SRA 1002 after its divergence from other citrus species. Further analyses will be conducted to determine the origin of these 25 TEs (new insertion events or derived from TEs already found in related citrus species) and their specific dynamics in AFL SRA 1002 and other Australian citrus species. The 25 consensus sequences were added to the citrus reference library of repeated sequences v2.0 [22].

**Figure 2:**
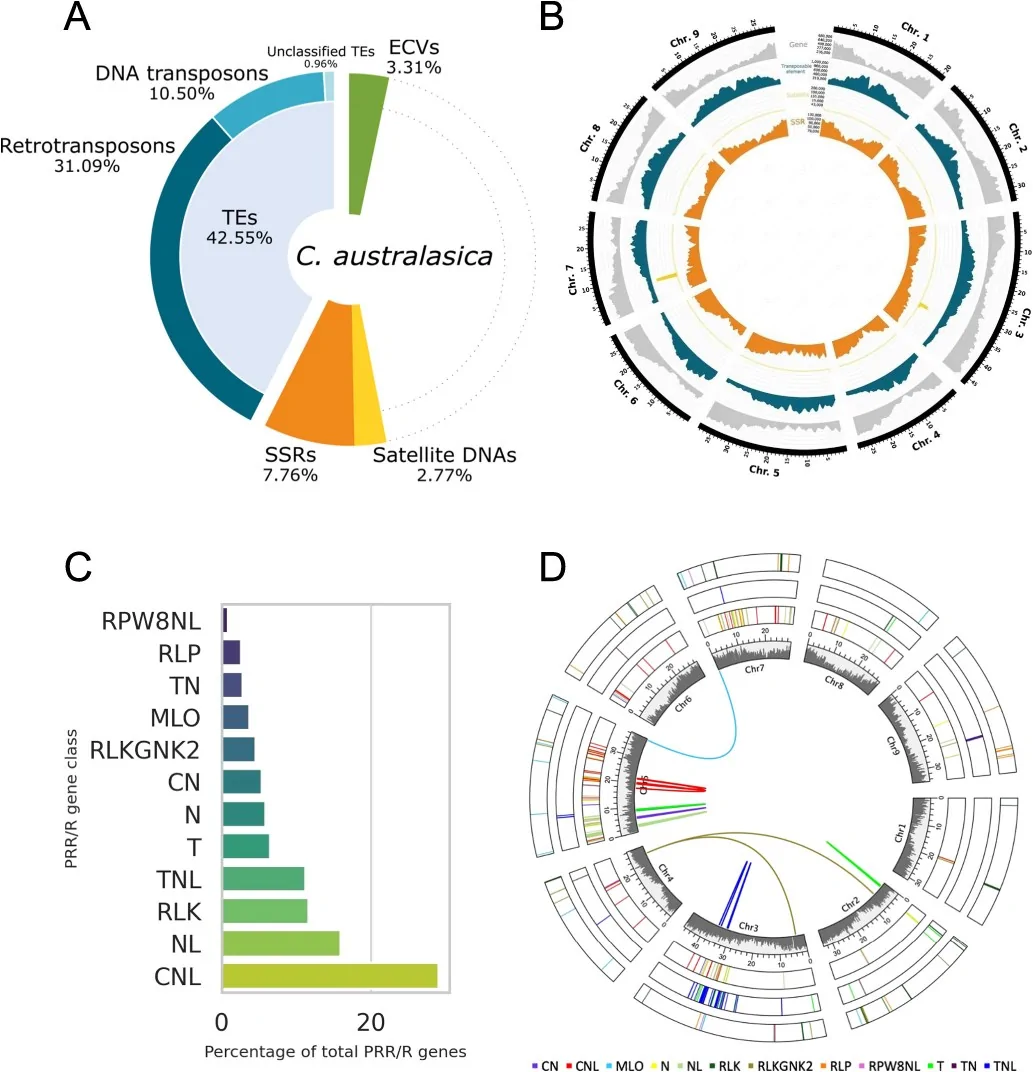
Annotation of repeated elements and immune receptor genes in the AFL SRA 1002 genome. (A) Composition of repeated elements across the genome, including retrotransposons (LTRs and LINEs), DNA transposons, SSRs, satellite DNAs, and ECVs. (B) Circos plot showing the chromosomal distribution of repeated elements in AFL SRA 1002. From outer to inner rings: (1) gene density; (2) TEs; (3) SSRs; (4) satellite DNAs; (5) ECVs. TEs are concentrated in centromeric and pericentromeric regions, while SSRs are enriched in gene-rich areas. (C) Proportional representation of 12 well-defined classes of pattern recognition receptors (PRRs) and resistance (R) genes annotated in the AFL SRA 1002 genome. (D) Chromosomal localization and class distribution of PRR and R genes across the 12 well-established classes. Each colored arc represents a gene of a specific PRR/R class mapped onto the nine pseudochromosomes of AFL SRA 1002. Colored lines in the center of the figure: duplication events. Grey histograms represent gene density.

**Table 2:** Estimated quantities of repeated sequences in the AFL SRA 1002 genome assembly.

	**Genome coverage (Mb)**	**Genome coverage (%)**
**Retrotransposons (Class I elements)**		
LTR Copia (RLC)	45.38	14.61
LTR Gypsy (RLG)	42.97	13.83
**LTR total**	**88.35**	**28.44**
LINE (RIX)	7.66	2.47
SINE (RSX)	0.53	0.17
Unclassified Class I	0.04	0.01
**DNA transposons (Class II elements)**		
TIR elements (DTX)	25.45	8.19
MITE	5.63	1.81
Helitron (DHX)	1.55	0.50
Unclassified_TE	2.98	0.96
**Total TE**	**132.18**	**42.55**
**ECVs**	10.27	3.31
**Satellite DNAs**	8.60	2.77
**SSR**	24.10	7.76
**Total repeated sequences**	**175.15**	**56.39**

Bold values indicate subtotal or total repeat categories.

As observed in other *Citrus* species, TEs were mainly concentrated in poor gene regions, including centromeres and pericentromeres, and formed highly concentrated chromosomal arms in acrocentric chromosomes such as Chr 5 to Chr 7 (Fig. 2B), also observed in Asian *Citrus* species [19]. Clusters of satellite DNAs, which covered 2.77% of the genome, were also identified in TE-rich regions (Chr 3 and Chr 7) and in telomeres (Chr 1, Chr 5, and Chr 8). On the contrary, simple sequence repeats (SSRs) were more concentrated in gene-rich regions and made up 7.76% of the genome. Finally, specific annotation allowed us to estimate that AFL SRA 1002 also contains a large number of endogenous caulimovirid elements (ECVs), representing 3.31% of its genome. The same diversity of ECVs has already been identified in other citrus species [23], suggesting the vertical inheritance of its elements during citrus speciation.

### Diversity of pattern recognition receptors

The annotation of the AFL SRA 1002 genome identified a total of 1280 genes related to PRRs and resistance (R) genes. Among them, 463 genes (36.1%) were classified into 12 well-established categories, excluding the Unknown class (Fig. 2C; Table 3). Together, the CNL, TNL, and NL classes represent the canonical intracellular NLR gene repertoire, totaling 260 genes in AFL SRA 1002. The CNL class was the most represented, accounting for 29.1% of the defined genes (135), followed by NL (74 genes, 15.9%), RLK (54 genes, 11.6%), and TNL (52 genes, 11.2%). Other classes included N (27 genes, 5.8%), RLKGNK2 (21, 4.5%), CN (25, 5.4%), T (29, 6.5%), TN (13, 2.8%), MLO (17, 3.7%), RLP (12, 2.6%), and RPW8NL (4, 0.9%). Comparable immune receptor repertoires reported in ancestral Asian citrus genomes and in Oceanian citrus species are summarized in Table S5, providing a comparative framework for interpreting pattern recognition receptors (PRR) and NLR diversity in AFL.

**Table 3:** Quantity of PRR and R genes present in AFL SRA 1002.

	**Class of PRR and R genes**													
	**CN**	**CNL**	**MLO**	**N**	**NL**	**RLK**	**RLKGNK2**	**RLP**	**RPW8NL**	**T**	**TN**	**TNL**	**UNKNOWN**	**Total**
**Quantity of PRR and R genes**	25	135	17	27	74	54	21	12	4	30	13	52	816	1280

Chromosomal mapping revealed a non-uniform distribution of PRR/R genes, with the highest densities observed on Chr 5 (124 genes), Chr 3 (116 genes), and Chr 7 (79 genes), and the lowest on Chr 4 (14), Chr 1 (15), and Chr 9 (19) (Fig. S2). These genes tended to cluster in gene-rich regions, particularly on Chr 2, Chr 3, and Chr 5, where the most represented classes—RLK, TNL, CNL, and NL—were especially abundant (Fig. 2D). Gene duplication analysis indicated several tandem duplication events, especially within the CNL and NL classes, each showing four duplication events (Fig. 2D). Notably, the genes Ciaus5g18130 and Ciaus5g16660 (CNL) and Ciaus4g25280 (NL) were each involved in two independent duplications, highlighting possible hotspots of immune gene expansion in the AFL genome (Table S6). By contrast, no duplication events were detected in the N, RLP, RPW8NL, and TN classes. These findings reveal a structurally and evolutionarily dynamic PRR/R gene repertoire, which could underlie specific immune adaptations in *C. australasica* and *C. australis*.

### Genomic mosaicism and structural variation reveal untapped diversity in Oceanian citrus

Comparative structural analysis between the newly assembled AFL SRA 1002 genome and the published genomes of *C*. *australasica* [24] and *C. australis* [15] revealed extensive conservation of syntenic blocks across all chromosomes, as well as multiple structural rearrangements such as inversions, translocations, and duplications (Fig. 3A). While the overall collinearity between AFL and both species was high, several large-scale discontinuities were observed, particularly in regions that failed to align or exhibited complex rearrangements, suggesting lineage-specific structural variation in AFL. These patterns highlight a mosaic genomic architecture in AFL, composed of large syntenic segments alternated with rearranged blocks, originating from both *C. australis* and *C. australasica* lineages. This structural configuration is consistent with an interspecific hybrid origin or a backcross scenario, as previously hypothesized from its nuclear mosaic signature and cytoplasmic inheritance.

**Figure 3:**
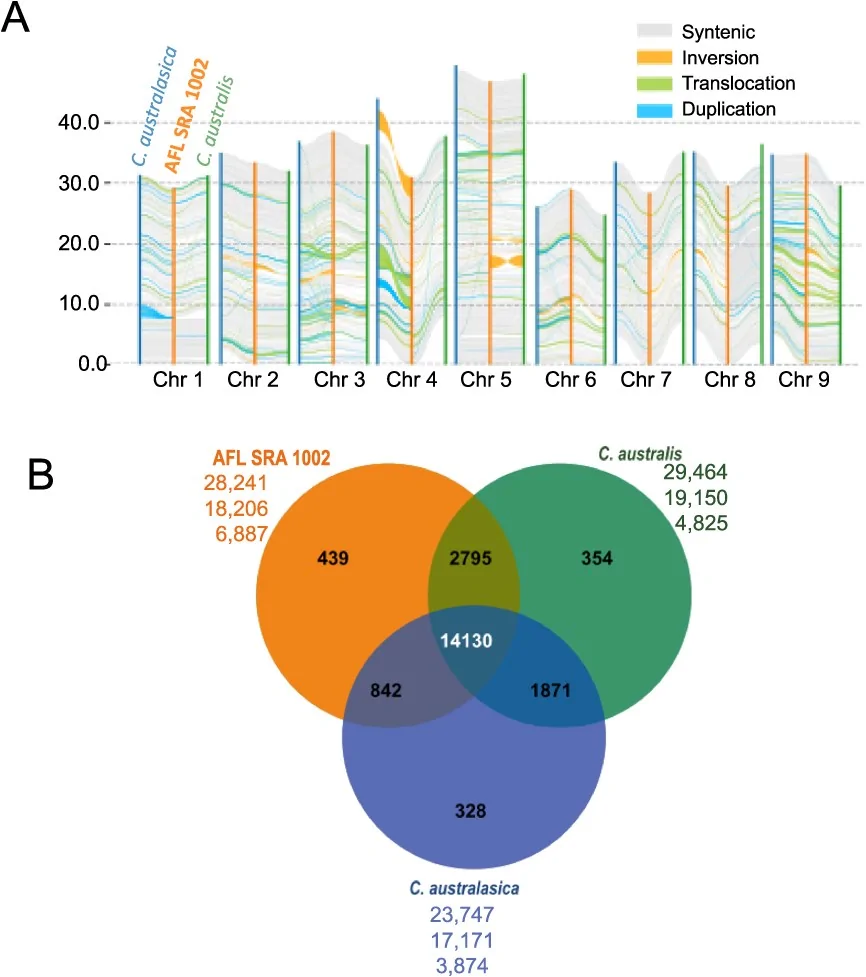
Structural genome comparison and gene clustering between AFL SRA 1002, *C. australasica*, and *C. australis.* (A) Genome-wide structural comparison between AFL SRR 1002, *C. australasica* (primary haplotype; [15]), and *C. australis* [14]. Conserved syntenic blocks are shown in grey; non-aligned regions appear in white. Structural rearrangements (inversions, translocations, duplications) are color-coded by type. Analysis was performed using SyRI. (B) OrthoMCL gene clustering among AFL SRR 1002, *C. australasica*, and *C. australis*. Venn diagram values represent the number of orthologous gene clusters. Below each species name, three values are shown: total number of genes, number clusters in each species, and number of unclustered genes.

Further comparisons with the two haplotype-resolved assemblies of *C*. *australasica* from Nakandala *et al.* [24] (Fig. S3) revealed asymmetric synteny between AFL and each haplotype, reinforcing the hypothesis of complex hybrid ancestry. Despite the overall conservation, AFL displayed a distinctive pattern of rearrangements that were not shared with either haplotype alone, which may reflect recombination or selection processes following interspecific admixture. Gene clustering analysis using OrthoMCL (Fig. 3B) supported these findings: of the 28 241 predicted chromosomal genes in AFL, 21 354 were assigned to 18 206 orthologous clusters. Most clusters included genes from *C. australasica* and/or *C. australis*, and 439 clusters were specific to AFL. The remaining 6877 genes did not cluster with any orthologs. These unclustered genes may correspond to lineage-specific sequences or highly diverged alleles not shared with the current *C. australasica* and *C. australis* references, or discrepancies arising from differences in genome assembly and gene annotation strategies. Their abundance, along with the observed structural variations, suggests a broader genetic diversity within Oceanian *Citrus* species than currently represented in available genome assemblies. These results indicate that additional diversity likely remains to be documented, particularly among undersampled or unsequenced Oceanian species accessions.

### Genome-wide diversity and phylogenetic relationships

Variant calling was performed from the WGS data of 132 citrus accessions (Table S7) using the AFL SRA 1002 reference genome. A total of 40 698 669 SNPs and 6 585 258 small indels were identified (Table 4). The SNPs were distributed relatively evenly across the nine pseudochromosomes, with Chr 3 showing the highest SNP count (6.61 million) and Chr 6 the lowest (3.79 million). The SNP density along the genome was also relatively homogeneous, however with a lower density in the first half of Chr 5 and the pericentromeric region of Chr 9 (Fig. S4).

**Table 4:** Total SNPs and indels per chromosome identified in 132 citrus accessions (filtering for less than 15% of missing data).

**Chromosome**	**SNPs**	**Indels**	**Total polymorphisms**
Chr 1	4 452 695	704 811	5 157 506
Chr 2	4 736 827	766 943	5 503 770
Chr 3	6 612 355	1 085 099	7 697 454
Chr 4	4 300 426	692 890	4 993 316
Chr 5	4 529 864	742 567	5 272 431
Chr 6	3 796 284	610 517	4 406 801
Chr 7	4 048 772	671 818	4 720 590
Chr 8	3 994 204	644 337	4 638 541
Chr 9	4 209 553	664 571	4 874 124
ChrUn	17 689	1705	19 394
**Total**	40 698 669	6 585 258	47 283 927

Analysis of heterozygosity along the genome reveals a unimodal distribution with a peak under 0.005 for all pure representatives of ancestral Asian and Oceanian species (Fig. S5A and B). It is also the case for the two *Citrus wakonai* [25] and of the accessions ‘*C*. sp. Cape York’ (unknown), discarding the possibility that they derive from recent interspecific hybridization (Fig. S5C). Among the other accessions, some display a unimodal distribution with a peak around 0.008 (mic-02fuD; supposed *C. australis × C. australasica* hybrid), 0.011 (oce-02fuD; PapCRC4111T; supposed *Citrus wintersii* Mabb. (1998) hybrids, Fig. S5D), and 0.017 (hEg-02fuD, hEg-orc2D and hEG-orfuD; supposed *C. glauca × C.* × *aurantium* L. (1753) hybrids, Fig. S5E), revealing their F1 interspecific status and an increasing divergence between parental species. The other accessions display multimodal heterozygosity distribution, suggesting complex admixture structures (Fig. S5F).

A filtered subset of 1 322 933 high-confidence diallelic SNPs covering the whole genome (with less than 5% missing data and ≥100 bp spacing) was used to build a nuclear phylogeny of the 103 accessions representative of ancestral species. The tree revealed a differentiation supported by a bootstrap value of 100% between two continental clusters with all Asian species (AsCl) on one side and all Oceanian (OcCl) the other side. Sub-branching within each continental cluster led us to identify 17 well-supported (bootstrap 100%) clades: 10 Oceanian and 7 Asian (Figs 4 and S6).

**Figure 4:**
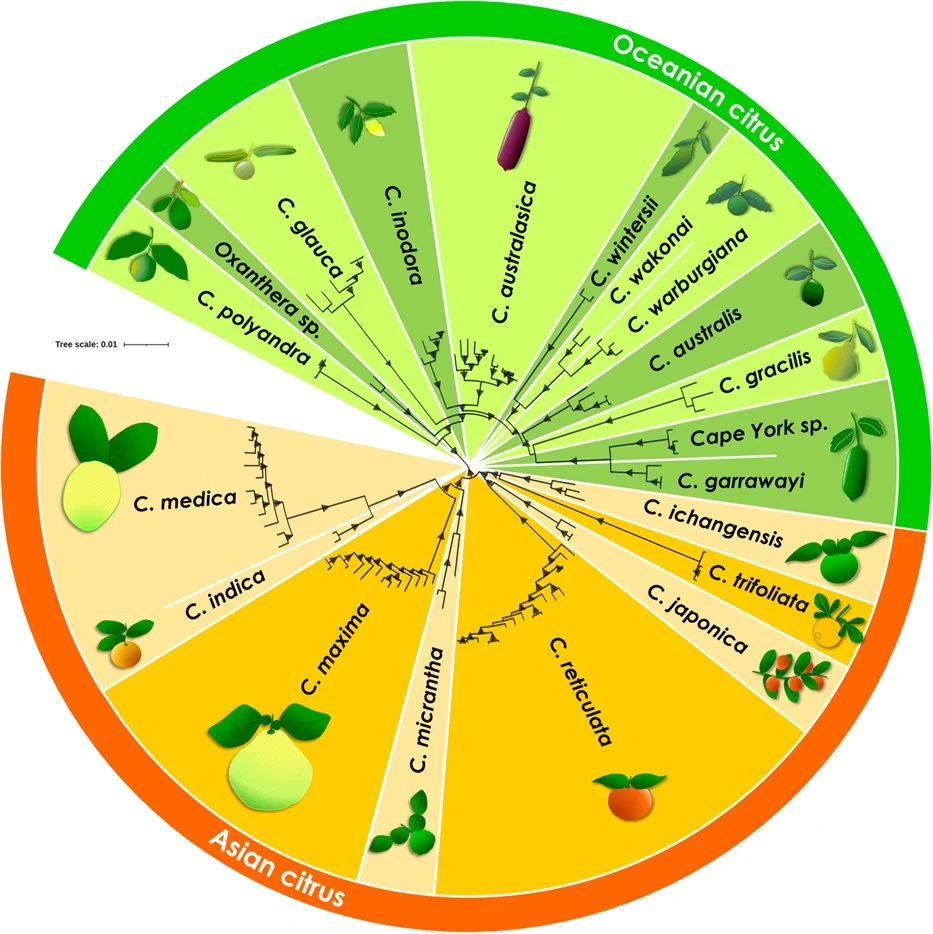
Nuclear phylogeny of 103 citrus accessions based on 1 322 933 genome-wide SNPs. The maximum likelihood tree identifies 17 well-supported clades, including 10 Oceanian lineages (green) and 7 Asian lineages (orange). A midpoint-rooted maximum likelihood tree was inferred using IQ-TREE v2.2.6 under the best-fit substitution model selected by ModelFinder Plus. Triangles indicate branch support (Boostrap >90; 1000 replicates), with size proportional to support values. The scale bar represents 0.01 substitutions per site.

*Citrus polyandra* (Ridel.) Burkill (1931) and *Oxanthera* Montrouz (1860) species, which are both highly homozygous (Fig. S5), constitute two of the Oceanian clades and are the most basal of the OcCl clade. *C. glauca* (desert lime, ADL) then joins OcCl and occupies a distinct and isolated position within the tree with a high differentiation. All species of the former *Microcitrus* genus [7] appear to be monophyletic. Within this ‘*Microcitrus*’ cluster, the three species from Papua New Guinea (*C. wintersii*, *Citrus warburgiana* F.M. Bailey (1901), and *C. wakonai*) constitute a monophyletic group that joins an Australian cluster constituted by *Citrus gracilis* Mabb. (1998) clade, *C. australis* clade, and a former clade joining *Citrus garrawayi* F.M. Bailey (1900) and the three unclassified accessions from Cape York (designated as Unknown in this paper). Among the Papua New Guinean species, *C. wakonai* and *C. warburgiana* appear to be closely allied at both nuclear and chloroplast levels (Fig. S7) and are included in a single main clade, while *C. wintersii* constitutes a second Papua New Guinea main clade. The AFL group (*C. australasica)* is supported as an independent lineage and a main clade of our analysis. It clusters with the last Oceanian main clade, *C. inodora,* with 100% bootstrap value.

For Asian species, seven main clades are distinguished with *Citrus medica* L. (1753) and *Citrus indica* Tanaka (1929), forming one of them at both nuclear and chloroplast levels. The rest of the organization is congruent with the previously published phylogeny of Asian citrus performed from WGS data [5, 26–28].

The chloroplast phylogeny, reconstructed using 3695 SNPs (missing data >5%) aligned to the *C.* × *sinensis* (L.) Osbeck (1765) plastid genome [29], fully confirmed the 10 Oceanian and 7 Asian main clades (Fig. S7). However, the tree structure is different from the nuclear one, as already described by Wu *et al.* [5] and Nakandala *et al.* [9]. These authors proposed that it may be explained by potential reticulate evolution, incomplete lineage sorting, or hybridization/introgression. More recently, Witharana *et al.* [30] extended the comparative nuclear versus chloroplast phylogenies using multiple alignment of each gene and concluded that incomplete lineage sorting predominantly drives these discordances, while ancient introgressions also contribute to topological discrepancies within certain clades.

Overall, the high-resolution nuclear phylogenetic reconstruction highlights a clear divergence between Oceanian and Asian citrus. It provides a robust framework for evolutionary and breeding studies.

### Diagnostic SNPs reveal clade differentiation and hybrid ancestry

To refine the resolution of diversity patterns, we identified DSNPs for the main Oceanian and Asian clades inferred from nuclear and chloroplast phylogenies. Ten Oceanian clades were considered (*C. wakonai* + *C. warburgiana* grouped; *C. garrawayi* + Unknown Cape York sp. treated jointly) and seven Asian clades (*C. medica* + *C. indica* grouped).

Across the 17 clades, 4 390 992 DSNPs were identified, with 2 617 304 in Oceanian and 1 689 010 in Asian clades (Table 5). Within Oceania, the highest DSNP counts occurred in *C. polyandra* (546 168), *C. gracilis* (489 820), *C. wintersii* (439 052), and *C. glauca* (411 918), while *C. australasica* showed the fewest (20 067), likely reflecting higher intraspecific diversity. DSNPs were distributed along all nine chromosomes, concentrated in gene-rich regions (Figs S8 and S9). Additionally, 84 678 DSNPs distinguished all Asian from all Oceanian accessions, providing a powerful genomic resource for tracing ancestry and lineage-specific traits (Table 5).

**Table 5:** DSNPs for the 17 main *Citrus* clades. Number of DSNPs identified per clade and chromosome.

	**Chr 1**	**Chr 2**	**Chr 3**	**Chr 4**	**Chr 5**	**Chr 6**	**Chr 7**	**Chr 8**	**Chr 9**	**Total**
**ADL**	50 589	51 316	71 980	48 994	37 345	44 101	35 674	37 700	34 219	411 918
**AFL**	2369	2619	3876	2483	672	1943	1444	2844	1817	20 067
**ARL**	14 670	13 408	16 264	14 037	3748	12 292	8429	8575	6778	98 201
**CLY**	59 984	67 997	93 170	61 131	55 223	53 891	54 341	52 882	47 549	546 168
**GAR/Un**	22 401	21 506	29 856	20 850	14 220	19 541	13 264	17 744	12 860	172 242
**GRA**	64 634	73 831	72 527	58 331	55 168	45 163	45 655	40 234	34 277	489 820
**INO**	7323	8072	12 074	8435	6833	6832	5377	7610	6929	69 485
**OXA**	20 972	23 484	31 933	21 623	19 544	19 745	18 872	20 100	17 889	194 162
**WAR/WAK**	20 943	23 205	29 034	21 601	14 350	19 460	15 614	15 965	16 017	176 189
**WIN**	49 491	58 555	69 793	56 237	38 151	41 755	42 947	41 034	41 089	439 052
**Total Oceanian**	**313 376**	**343 993**	**430 507**	**313 722**	**245 254**	**264 723**	**241 617**	**244 688**	**219 424**	**2 617 304**
**ICH**	10 992	12 364	15 135	10 697	7639	10 990	7339	8425	7844	91 425
**JAP**	19 429	20 368	29 342	20 849	16 326	18 448	15 084	17 049	12 650	169 545
**MAX**	14 871	15 202	20 329	14 187	10 974	13 270	11 517	10 558	9531	120 439
**MED/IND**	29 791	34 580	44 807	31 879	28 119	27 076	27 299	23 916	24 684	272 151
**MIC**	9175	11 259	14 967	10 771	8545	10 033	8200	8944	7043	88 937
**RET**	20 354	21 060	29 189	19 135	15 915	17 991	13 922	15 829	13 776	167 171
**TRI**	87 199	100 508	132 142	88 793	75 223	78 312	72 359	75 099	69 707	779 342
**Total Asian**	**191 811**	**215 341**	**285 911**	**196 311**	**162 741**	**176 120**	**155 720**	**159 820**	**145 235**	**1 689 010**
**Asian/Oceanian**	10 116	10 765	14 942	10 481	8512	8076	7405	8065	6316	84 678
**Total**	**515 303**	**570 099**	**731 360**	**520 514**	**416 507**	**448 919**	**404 742**	**412 573**	**370 975**	**4 390 992**

Bold values correspond to cumulative values calculated for taxonomic groups and for the complete dataset.

Species within shared main clades also displayed specific DSNPs: *C. warburgiana* + *C. wakonai* (93 807 and 85 026 DSNPs), *C. garrawayi* + Unknown Cape York sp. (65 420 and 100 055), and *C. medica* + *C. indica* (239 123 and 133 783). Their genome-wide distribution (Table S8; Fig. S10) supports the validity of these taxa, consistent with their low heterozygosity and the chloroplast phylogeny.

Using the AFL reference genome as a mapping backbone, DSNPs refined phylogenomic resolution and enabled the detection of hybrid ancestry. Among Oceanian species, interspecific introgressions were detected only in *C. australasica*, where four accessions, including SRA 1002, displayed heterozygous *C. australasica*/*C. australis* regions (Fig. S11), all sharing the *C. australasica* chlorotype (Fig. S12). These *C. australasica*/*C. australis* admixtures correspond to *C. × virgata* [31], as proposed by Mabberley [1], encompassing accessions from Corsica and Brazil that exhibit complex hybrid structures consistent with selfing or backcrosses to *C. australis* (Fig. 5A).

**Figure 5:**
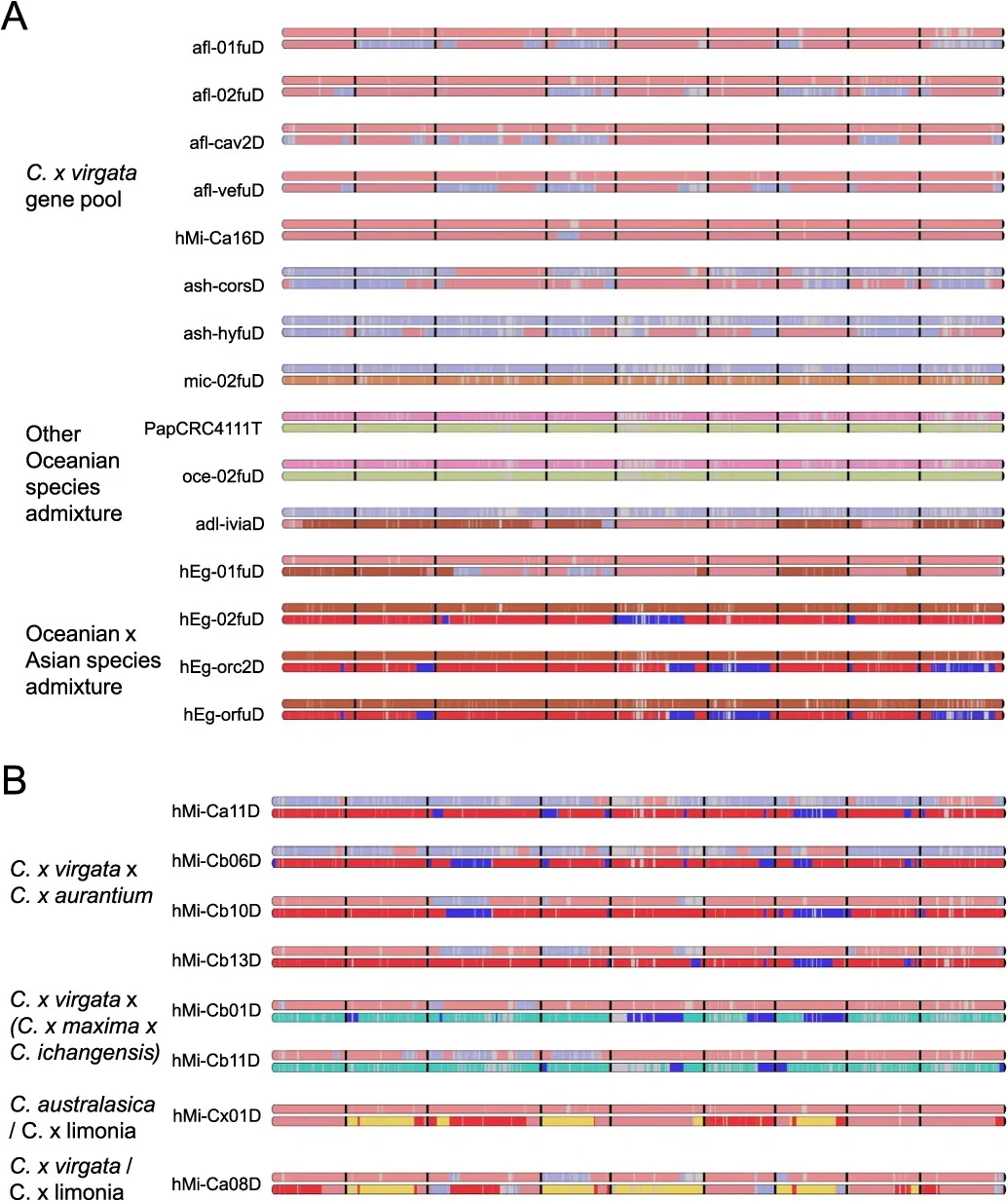
Phylogenomic mosaic structure along the nine chromosome (separated by black dash). (A) Admixed Oceanian citrus germplasm. (B) Aleatory admixed Oceanian/Asian hybrids of the CIRAD/INRAE breeding program. Color code: salmon: *C. australasica;* light blue: *C. australis,* light brown: *C. inodora*; green: *C. warburgiana*; pink: *C. wintersii*; dark brown: *C. glauca;* red: *C. reticulata*; blue: *C. maxima;* cyan: *C. ichangensis;* yellow: *C. medica.*

Other admixed genotypes included an F₁ *C. australis × C. inodora* (mic-02FuD), two F₁ *C. wintersii × C. warburgiana* (PapCRC411T, oce-02FuD), and complex mosaics combining *C. australasica*, *C. australis*, and *C. glauca* (adl-iviaD, hEg-01FuD), each with chlorotypes matching the maternal species (Fig. S12). Eremorange hybrids (*C. glauca × C. × aurantium*) showed expected genomic mosaics of *C. glauca* and *C. reticulata*/*Citrus maxima*, with identical structures across Corsican and Brazilian accessions, confirming their identity (Fig. S12). Asian admixed species exhibited mosaic genomic architectures consistent with previous analyses using Asian references [19] (Fig. S13).

Further analyses of greenhouse-derived hybrids from random Oceanian *×* Asian pollinations revealed various generations of admixture (Fig. 5B). Four hybrids appear as F₁ between *C. × virgata* and *C. × aurantium* (all *C. reticulata* chlorotype), two as F₁ combinations of Oceanian and Asian genomes (*C. australasica*, *C. australis*, *Citrus ichangensis*, *C. maxima*; *C. australasica* chlorotype), and two advanced-generation mosaics likely involving *C. × limonia* (Asian side) and *C. australasica* or *C. × virgata* (Oceanian side), both with *C. reticulata* chlorotype (Fig. S12).

Altogether, these results demonstrate that DSNPs not only delineate clades with high accuracy but also allow precise reconstruction of hybridization and introgression patterns, supporting germplasm authentication and guiding breeding strategies (Fig. 5A and B).

### Gene family evolution reveals functional reorganization between Asian and Oceanian citrus lineages

We investigated gene family evolution and functional reorganization by comparing AFL SRA 1002 with four ancestral Asian citrus taxa [19], using genomes consistently annotated with the EuGene pipeline [32]. Orthologous groups were inferred using OrthoFinder [33, 34], followed by gene family evolution modeling with CAFE [35], using the species phylogeny derived from genome-wide variation as a constraint (Fig. 4).

**Figure 6:**
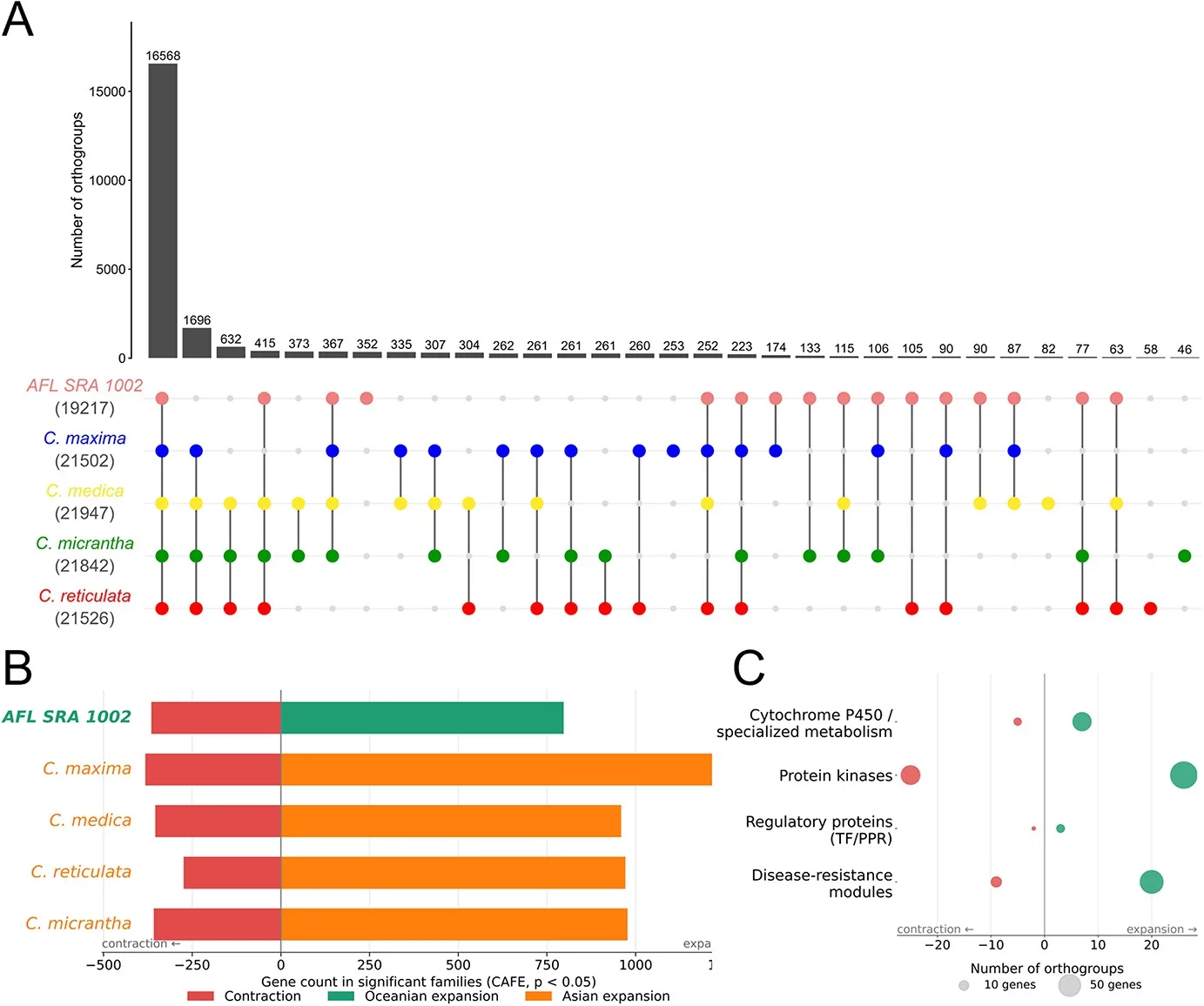
Gene family dynamics and functional reorganization in AFL SRA 1002 compared to Asian citrus lineages. (A) Distribution of orthogroups across five citrus genomes inferred using OrthoFinder. The majority of orthogroups are shared across all species, with smaller subsets of lineage-specific and partially shared gene families. Detailed intersection patterns are shown in Fig. S15. (B) Number of significantly expanded and contracted genes inferred using CAFE. Bars represent the number of expanded (right) and contracted (left) genes for each species. AFL SRA 1002 shows a pronounced expansion bias compared to Asian citrus species. (C) Functional distribution of significantly evolving gene families in AFL SRA 1002. Each point represents a functional category associated with expanded (green) or contracted (red) orthogroups. The *x*-axis indicates the number of orthogroups, and point size reflects the number of genes involved.

Gene family clustering revealed a highly dominant conserved core of orthogroups shared across all five genomes (16 568 orthogroups), reflecting strong conservation of gene content across citrus lineages (Fig. 6A). Beyond this core, the distribution of partially shared orthogroups was highly structured and non-random, with a long tail of low-frequency combinations. AFL SRA 1002 showed a distinct participation pattern within these intersections. While consistently present in the conserved core, AFL was less frequently involved in large, cohesive subsets shared exclusively among Asian genomes and instead contributed to a broader spectrum of smaller, fragmented orthogroup combinations. These included multiple low-frequency intersections involving AFL and one or a few Asian taxa, suggesting differential gene retention and lineage-specific diversification (see detailed intersections in Figure S14). In addition, AFL displayed a set of private orthogroups, further supporting the presence of a lineage-specific gene repertoire. Altogether, these patterns indicate that, although AFL shares a conserved backbone with Asian citrus genomes, its gene content is reorganized through a combination of specific gene gains, losses, and differential retention, consistent with its evolutionary divergence within the Oceanian lineage.

Gene family evolution analysis revealed lineage-specific expansion–contraction patterns, without evidence of a simple AFL-driven expansion trend (Fig. 6B). Although all genomes exhibited substantial numbers of genes in expanded families, with *C. maxima* showing the strongest expansion signal, AFL displayed a more balanced profile, with both expansions (+797 genes) and contractions (−365 genes) contributing to its gene family dynamics. In contrast, Asian species consistently showed a stronger expansion bias relative to contraction. Functional characterization of significantly evolving gene families in AFL SRA 1002 revealed a structured pattern of gene family remodeling across major functional categories (Fig. 6C). Rather than showing uniform directional shifts, protein kinases exhibited the strongest turnover, with substantial numbers of both expanded and contracted orthogroups, but a higher number of genes involved in expansions, indicating amplification within specific subfamilies.

Disease-resistance modules showed a clear bias toward expansion, with both more orthogroups and a larger number of genes on the expansion side, suggesting reinforcement of specific components of the defense repertoire. Similarly, cytochrome P450/specialized metabolism displayed a consistent expansion-oriented pattern, indicative of selective amplification of metabolic gene families. In contrast, regulatory proteins (TF/PPR) showed more limited changes, with a slight predominance of expansion but overall lower numbers of orthogroups and genes involved. Overall, these results indicate that gene family evolution in AFL SRA 1002 is not driven by uniform shifts across functional categories, but rather by selective remodeling within gene families. While metabolic and defense-related functions show clearer expansion trends, signaling-related components, particularly protein kinases, display strong bidirectional turnover, consistent with functional specialization and lineage-specific adaptation.

## Discussion

In this study, we report a high-quality chromosome-scale genome assembly of the AFL SRA 1002, a backcross hybrid *C. australasica ×* (*C. australasica × C. australis*) *with C. australasica* cytoplasm. The assembly integrates long-read Nanopore sequencing, PCR-free Illumina reads, optical mapping, and a high-density interspecific genetic map, resulting in nine pseudomolecules spanning over 97% of the estimated genome. The AFL SRA 1002 assembly shows very high synteny and collinearity with the *C. australis × C. inodora* genetic map and the consensus *Citrus* map [20], confirming strong structural conservation across the genus. It reveals a high degree of synteny and collinearity of our assembly with other citrus genomes, such as *C. reticulata* [19]. Comparative analyses with recently published genomes of *C. australis* [14] and other two *C. australasica* [15, 24] accessions also confirm strong synteny across Oceanian citrus despite their ecological divergence.

Compared to previously published genomes of pure *C. australasica* and *C. australis* accessions [14, 15, 24], the SRA 1002 assembly represents a valuable hybrid genome that may reflect the recombined genetic mosaic present in breeding germplasm. The AFL SRA 1002 genome is therefore well suited for population-level analyses, QTL mapping, and candidate gene identification in interspecific hybrids aimed at resistance introgression.

Segregation distortion in the genetic map, particularly the counter-selection of *C. australis*-derived regions on Chr 1, Chr 3, and Chr 5, indicates postzygotic selection or gametic incompatibility, which could limit the recovery of certain hybrid genotypes. Understanding these transmission biases will be useful for guiding controlled hybridizations among Oceanian species.

Annotation revealed a TE landscape (42.55% of the genome) broadly consistent with other citrus genomes, but with distinct lineage-specific activity. Twenty-five new TE consensus sequences were found, mainly LTRs and LINEs, highlighting active or ancient transpositional bursts in Oceanian lineages. Future comparative annotation of TEs and ECVs across Australian species will help assess their contribution to genome evolution and gene regulation. The immune receptor repertoire (1280 PRR and R genes) shows both conservation and expansion of major resistance classes, with CNLs, NLs, and TNLs predominating. The total number of NLRs (261) is lower than in *C. australasica*, *C. glauca*, and *C. inodora* [15], which range from 319 to 576 NLR genes depending on the species and haplotype (Table S5). However, the relative distribution among NLR subclasses remains similar, with CNL and TNL classes dominating the repertoire. Differences in total NLR counts likely reflect both biological variation among accessions and methodological differences in gene identification pipelines. Interestingly, major clusters of resistance genes occur on Chr 3, Chr 5, and Chr 7 in AFL, a distribution also reported in ancestral Asian citrus genomes (*C. reticulata*, *C. maxima*, *C. medica*, and *C. micrantha*, [19]) as well as in Oceanian citrus genomes such as *C. australasica*, *C. glauca*, and *C. inodora* [15], suggesting a conserved genomic organization of immune receptor loci across citrus lineages. The conservation of these resistance gene clusters across citrus genomes likely reflects shared evolutionary hotspots for immune receptor diversification. However, the functional contribution of these loci to disease tolerance remains unclear, particularly given that enhanced tolerance to *Clas* has primarily been reported in Oceanian citrus species. The AFL SRA 1002 assembly therefore provides an additional genomic resource from an interspecific Oceanian hybrid background, offering a useful framework to investigate the evolutionary dynamics and functional diversification of immune receptor loci in citrus. Importantly, AFL SRA 1002, from the Corsican citrus collection, is a confirmed interspecific hybrid, whereas Singh *et al.* [15] analyzed pure accessions from the Givaudan Citrus Variety Collection at the University of California Riverside. The enrichment of tandem duplications within the CNL and NL classes suggests ongoing diversification of defense loci, making these clusters good candidates for future functional studies, particularly in relation to *Clas* response.

While recent efforts have produced high-quality genome assemblies for Oceanian lime species [36], our study builds on these resources to address diversity and lineage structure at the population scale. Using the AFL genome as a reference, we resequenced other 54 Oceanian citrus accessions. These new data were joined with 78 previously published WGS resequencing data to perform a variant calling identifying close to 42 million SNPs. Among them, a subset of more than 1.3 million high-confidence SNPs was used to establish a high-resolution phylogenomic reconstruction, resolving lineage structure across Oceanian citrus. While previous studies based on plastomes and limited nuclear markers [9] supported the monophyly of Australian citrus, our use of whole-genome resequencing and availability of Papua New Guinea and New Caledonia species significantly expands this perspective. We demonstrated the monophyly of all Oceanian species and identified 84 678 DSNPs that fully differentiated all Oceanian species from all Asian ones.

*C. polyandra* appears as the basal species of the Oceanian clade. From a chemotaxonomy perspective, a study proposed that *C. polyandra* may be a *Citrus japonica* Thunb. (1780) hybrid [37]. The strong homozygosity of *C. polyandra* and the important differentiation of *C. polyandra* from all other *Citrus* species definitively discard this hypothesis.

The two species of the former *Oxanthera* genus cluster together, constituting a clade with a large differentiation with other Oceanian species. As *C. polyandra*, they cluster basal to the remaining Oceanian lineages, confirming their inclusion in the *Citrus* genus as true species and reinforcing their unique evolutionary trajectory.

Interestingly, all the species from the former *Microcitrus* genus [7] constitute a monophyletic group differentiated from the *C. glauca* clade (former *Eremocitrus* genus). This differentiation of *C. glauca* is consistent with an earlier evolutionary divergence and adaptation to a significantly harsher climate. *C. glauca* is also known for its strict resistance to HLB [10]. Within the *Microcitrus* group, we reveal the monophyletic origin of the three Papua New Guinea species (*C. wintersii*, *C. warburgiana*, and *C. wakonai*). The close relationship between *C. warburgiana* and *C. wakonai*, a new species endemic of Goodenough Island, recently discovered by Forster and Smith [25] is observed in both nuclear and chloroplast phylogeny; however, the high homozygosity of *C. wakonai* and the identification of DSNPs for *C. wakonai* and *C. warburgiana* all along the genome validate *C. wakonai* as an independent species. The Papua New Guinea species clusters with a group of Australian species, including *C. gracilis*, *C. australis*, and *C. garrawayi* and the three unclassified ‘Cape York’ accessions. We confirm the relatedness of *C.* sp Cape York accessions and *C. garrawayi* already observed in a previous study [9], while the important homozygosity and the identification of DSNPs all along the genome support the ‘*C.* sp Cape York’ group, as a distinct morphology than *C. garrawayi*, as a new species. *C. australasica* clade, which is of considerable commercial interest due to its described resistance/tolerance to HLB and culinary value, and *C. inodora* clade constitute a second big cluster of the species of the former *Microcitrus* genus.

From resequencing data of the 103 representatives of ancestral species, we developed a robust panel of 4 390 992 DSNPs capable of tracing the genomic origin of any citrus accession, including hybrids. This DSNPs panel is effective to distinguish, respectively, 12 and 8 Oceanian species.

This tool allows precise clade assignment and admixture detection, with broad applications in germplasm curation, taxonomic clarification, and breeding program design, as we have illustrated with the identification of admixed accessions in the germplasm collections of the partners and admixed hybrids from uncontrolled crosses. The identification of interspecific F1 hybrids and admixed accessions between Oceanian species in germplasm collections suggests a huge potential for interspecific hybridization between these monoembryonic non-apomictic species. Many of these hybridizations may have occurred *ex situ* during germplasm exchanges or propagation by seeds, but the *in situ* vicinity of species may have resulted in wild interspecific hybridization. It may be the case for the introgression of *C. australis* in finger limes as, for example, wild accessions of both species have been described in close locations between Brisbane and Gold Coast.

Unlike traditional DNA markers, the DSNP panel enables unambiguous assignment of accessions to their ancestral clades all along the genome using genome-wide allelic signals. Beyond refining phylogenetic resolution, this approach enables the accurate identification of hybrid ancestries and varietal origins across global citrus, offering a transformative framework for germplasm classification and utilization. It is also a highly valuable tool for GWAS analysis based on ancestral haplotype and the driving of interspecific introgression schemes. This scalable resource represents a major advance for citrus diversity research and applied breeding.

Kasp markers developed from DSNPs well dispersed in the genome were used to decipher meiotic behavior and establish genetic maps of tetraploid limes [38], citrumelo and citrange [39] from triploid and tetraploid progenies, respectively. Such markers are also routinely used to differentiate zygotic and nucellar seedlings for most citrus rootstocks that result from F1 interspecific hybridization [40, 41].

Using high-throughput genotyping (GBS and WGS), DSNPs have enabled to demonstrate *C. maxima* introgression in most cultivated mandarins [5, 42] and more broadly to decipher interspecific mosaic of cultivated *Citrus* [5, 19, 43, 44]. GBS data also have allowed to analyze the meiotic behaviors of tetraploid interspecific parents, to establish genetic maps and perform association studies in tetraploid progenies, aiming to select new citrus rootstocks [45]. In the absence of a citrus reference panel for genotype imputation from low-coverage sequencing data, DSNPs provide the possibility to trace ancestral genome fragments in segregating progenies from interspecific hybrids. In a similar way that we used WGS data of admixed individuals resulting from random interspecific hybridization over several generations (Fig. 5B), we currently use this approach for ultra-high-density genetic mapping, based on the location of interspecific crossovers, from interspecific backcross-like progenies.

Gene family evolution analysis based on orthology inference and phylogeny-aware modeling provides a robust framework to interpret gene content variation across citrus lineages. Using an orthology-based approach (OrthoFinder) combined with gene family evolution modeling (CAFE), we identified contrasted lineage-specific patterns of gene family expansion and contraction between Asian and Oceanian citrus. Rather than indicating a simple directional trend, AFL SRA 1002 exhibited a balanced dynamic, with substantial contributions of both gene gains and losses, consistent with the absence of a global expansion bias. Functional characterization of significantly evolving gene families revealed a non-random pattern of genomic remodeling. Gene families associated with signaling and regulatory processes, particularly protein kinases, displayed strong bidirectional turnover, indicating dynamic restructuring of signaling components rather than systematic contraction. Similarly, defense-related gene families, including components of receptor-mediated immunity, showed a mixed pattern, with selective expansion of specific subfamilies alongside contraction of others, consistent with the structured but non-uniform PRR/R repertoire observed in AFL. In contrast, expanded gene families were predominantly enriched in cytochrome P450-related functions and other components of specialized metabolism, together with subsets of defense-related genes. This pattern is consistent with previous genomic studies of Australian citrus, which reported lineage-specific enrichment of genes involved in plant–pathogen interactions, stress responses, and specialized metabolic pathways [8, 24]. Taken together, these results indicate that gene family evolution in Oceanian citrus is not driven by uniform expansion or contraction across functional categories, but rather by selective remodeling within gene networks. The combined evidence from gene family evolution and PRR/R gene distribution supports a model in which Oceanian citrus exhibit functional reorganization of defense and signaling systems, characterized by targeted expansion of metabolic and defense-related functions alongside dynamic turnover of signaling components. Such a configuration may reflect alternative adaptive strategies, where responses to environmental stress and pathogens rely on a combination of specialized metabolism and flexible signaling architectures rather than extensive diversification of canonical immune receptors. This interpretation is consistent with the reported tolerance to HLB in several Australian citrus species, although the underlying molecular mechanisms remain to be elucidated.

Nevertheless, the AFL SRA 1002 genome is integrated into the Citrus Genome Hub (https://citrus-genome-hub.southgreen.fr/), which provides a genome browser and several user-friendly tools for molecular analyses. Beyond phylogenetics, the SNP dataset generated provides a foundation for genome-wide association studies, selection scans, and trait mapping. Combined with the AFL reference genome, this framework represents the most comprehensive genomic toolkit to date for Oceanian citrus. It enables the strategic integration of wild diversity into breeding pipelines, particularly for traits like HLB resistance, environmental resilience, and fruit quality, and will support both fundamental research and global citrus improvement efforts.

## Conclusion

This study presents a high-quality, chromosome-scale genome assembly of *C. australasica* AFL SRA 1002 and the most extensive genomic survey to date of Oceanian citrus diversity. By integrating *de novo* assembly, genome annotation, and high-depth resequencing data of 132 accessions, we provide a robust framework for understanding the evolutionary history, structural variation, and gene content of this underexplored citrus lineage. The validation of novel species, the development of species diagnostic SNPs, and the identification of lineage-specific genes collectively underscore the value of Oceanian citrus as a reservoir of adaptive diversity. These resources lay the groundwork for functional and evolutionary studies and targeted breeding strategies aimed at enhancing stress tolerance, disease resistance, and fruit quality in cultivated citrus. As global threats such as HLB intensify, the integration of wild diversity through genomics-informed approaches will be essential for sustaining citrus production and driving innovation in the years to come.

## Materials and methods

### Genomic sequences of citrus accessions

#### Accession used for sequencing and optical mapping for de novo assembly

The genome assembly of the AFL was generated from accession SRA 1002, maintained in the Corsican field collection of the Citrus Biological Resource Center (Citrus BCR, INRAE-Cirad, San Giuliano, Corsica, France [17]).

#### Resequencing of citrus accessions

For the diversity analyses, 54 citrus accessions were newly resequenced and combined with 78 publicly available datasets from NCBI and EBI (Table S7). The 54 newly sequenced accessions were selected to expand the coverage of Oceanian citrus diversity across taxa and geographic sources, prioritizing (when available) accessions with minimal admixture signals in prior screening and complementing the set of 78 publicly available genomes used here. This set of new accessions allowed us to cover all Oceanian *Citrus* species from Australia (*C. australasica*, *C. australis*, *C. glauca*, *C. garrawayi, C. gracilis*, and *C. inodora*) and Papua New Guinea (*C. wakonai*, *C. warburgiana*, *C. wintersii*, and *C. polyandra*), as well as two species of the former *Oxanthera* genus from New Caledonia. Among the 54 new accessions, 18 were provided by the Bundaberg Research Station (Queensland Australia), 14 by Fundecitrus (Sao Paulo state, Brazil), 11 by Cirad-INRAE breeding program (Corsica, France), 6 by IVIA (Valencia, Spain), 3 by the Corsican field collection hosted by the Citrus BRC INRAE-Cirad (Corsica, France), and 2 by IAC (New Caledonia).

Genomic DNA was extracted using the DNeasy Plant Mini Kit (Qiagen). Two library types were used: (i) standard PCR-free Illumina libraries prepared for 33 accessions and sequenced on a HiSeq 4000 platform and (ii) Tell-Seq linked-read libraries prepared for 21 accessions and sequenced on an Illumina NovaSeq platform, both with an average coverage of ~30× per accession. R1 and R2 reads from Tell-Seq datasets were used for variant calling.

Demultiplexing, quality filtering, and adapter removal followed standard Genoscope and Montpellier GenomiX protocols. All datasets were processed using the same downstream pipeline to ensure comparability across sequencing technologies.

### Variant calling from resequencing data

Variant calling was performed using the Snakemake pipeline developed by Droc *et al.* [19], which automates GATK4 best-practice workflows for large-scale genomic analyses. Cleaned reads (fastp v0.20.1, [46]) from 54 newly sequenced accessions were combined with 78 publicly available datasets from EBI and NCBI (Table S5). Reads were aligned to the new finger lime reference genome using BWA, and SNP/indel calling followed the GATK4 standard pipeline, including base quality recalibration and joint genotyping steps (GenomicsDBImport → GenotypeGVCFs). This unified workflow ensured consistent variant discovery and reproducibility across all datasets.

### Comparative genomic analysis and gene family identification

To assess synteny and structural divergence between haplotypes, SyRI v1.3 [47] was used. Prior to this, the genome assemblies were aligned using nucmer from MUMmer v4.0.4 with the parameters --maxmatch -l 100 -c 500 -b 500, and filtered with delta-filter (-m -I 70 -l 100) [48]. For gene family identification, we used a comparative analysis to examine the conservation of gene repertoires among orthologs in the genomes of AFL SRA 1002, *C. australasica* [15], and *C. australis* [24]. First, we aligned all-to-all proteins using BLASTP (e-value of 1e−5). Genes were then clustered using OrthoMCL v1.4 [49] implemented in OrthoVenn3 [50] with a Markov inflation index of 1.5 and a minimum e-value of 1e−15.

### Admixture analyses

DSNPs were identified following the two-step method of Ahmed *et al.* [43] to discriminate ten Oceanian and seven Asian main clades, plus six species grouped within three composite clades. Analyses were based on 48 Oceanian and 55 Asian accessions (Table S5). Briefly, SNPs with GST ≥ 0.5 were first used to detect introgressed regions, which were then excluded before a second run selecting fully diagnostic loci (GST = 1). Detailed procedures are provided in Ahmed *et al*. [43], and Supplementary Note Note  2 and Figure S15.

### Phylogenetic inference from nuclear and chloroplast SNPs

Maximum likelihood phylogenetic trees were reconstructed using IQ-TREE v2.2.6 [51] based on aligned SNP datasets derived from nuclear and chloroplast genomes. The nuclear SNP alignment comprised 103 accessions and was built from a high-confidence set of concatenated loci, while the chloroplast SNP alignment included 100 accessions. For both datasets, the best-fit substitution model was selected using ModelFinder Plus (-m MFP), and branch support was assessed using 1000 ultrafast bootstrap replicates (-B 1000) and 1000 SH-aLRT replicates (-alrt 1000). The resulting trees were midpoint-rooted using Biopython v1.83 and exported in Newick format for downstream analyses and visualization.

### Gene family evolution analysis

Comparative analyses were conducted using chromosome-scale genome assemblies from Asian citrus species [19] and AFL SRA 899 genome generated in this study. For each species, only the longest protein isoform per gene was retained. Orthologous gene families were identified using OrthoFinder v2.5.2 [33, 34] with default parameters. Functional annotation of orthogroups was conducted by extracting gene-level annotations from the GFF3 files of each genome and integrating these annotations across all genes within each orthogroup to assign a consensus functional description. The species phylogeny used for gene family evolution analyses was derived from the genome-wide tree described in the section *‘*Genome-wide diversity and phylogenetic relationships’ (Fig. 4). This topology was used as a constraint in CAFE v5.0.0 [35], which re-estimates branch lengths based on gene family size variation under a birth–death model. This procedure generates a tree specifically optimized for modeling gene gain and loss while maintaining the evolutionary relationships inferred from genome-wide data. Expanded and contracted gene families were functionally interpreted based on orthogroup annotations and grouped into major biological categories (e.g. signaling, metabolism, and plant–pathogen interaction, Table S9).

## Supplementary Material

Web_Material_uhag213

## Data Availability

Raw data from long-read sequencing and genome assemblies of *C. australasica* AFL SRA 1002 are available at EBI under the bioprojects PRJEB78475 and PRJEB78536. RNAseq data used for genome annotation are available at EBI under the bioproject PRJEB104880. Raw resequencing data of the new 54 citrus accessions are available at EBI under the bioprojects PRJEB97093 (WGS) and PRJEB103773 (Tellseq).

## References

[1] Mabberley DJ . A classification for edible citrus: an update, with a note on *Murraya*. Telopea. 2022;25:271–84

[2] Ollitrault P, Curk F, Krueger R. Citrus taxonomy. In: Talon M, Gmitter FG, Caruso M (eds.), The Genus Citrus. Elsevier, 2020, 57–81.

[3] Wang X, Xu Y, Zhang S. et al. Genomic analyses of primitive, wild and cultivated citrus provide insights into asexual reproduction. Nat Genet. 2017;49:765–72 10.1038/ng.3839 28394353

[4] Curk F, Francois L, Hussain S. et al. Citrus origins. In: Citrus Production: Technological Advancements and Adaptation to Changing Climate, (1st ed.). Boca Raton: CRC Press, 2022

[5] Wu GA, Terol J, Ibanez V. et al. Genomics of the origin and evolution of citrus. Nature. 2018;554:311–6 10.1038/nature25447 29414943

[6] Ghosh D, Kokane S, Savita BK. et al. Huanglongbing pandemic: current challenges and emerging management strategies. Plants. 2022;12:160 10.3390/plants12010160 PMC9824665 36616289

[7] Swingle WT, Reece PC. The botany of Citrus and its wild relatives. In: Reuther W, Batchelor LD, Webber JJ (eds.), The Citrus Industry. Berkely: University of California Press, 1967, 190–430.

[8] Nakandala U, Furtado A, Masouleh AK. et al. The genomes of Australian wild limes. Plant Mol Biol. 2024a;114:102 10.1007/s11103-024-01502-4 PMC11422456 39316221

[9] Nakandala U, Furtado A, Smith MW. et al. Phylogenetic relationships among Australian native citrus species based upon complete chloroplast genomes and single copy nuclear genes. Trop Plants. 2023a;2:00–21

[10] Alves MN, Lopes SA, Raiol-Junior LL. et al. Resistance to ‘*Candidatus Liberibacter asiaticus*’, the Huanglongbing-associated bacterium, in sexually and/or graft-compatible citrus relatives. Front Plant Sci. 2021;11:617664 10.3389/fpls.2020.617664 PMC7820388 33488659

[11] Ramadugu C, Keremane ML, Halbert SE. et al. Long-term field evaluation reveals Huanglongbing resistance in citrus relatives. Plant Dis. 2016;100:1858–69 10.1094/PDIS-03-16-0271-RE 30682983

[12] Mahmoud LM, Dutt M. Novel citrus hybrids incorporating Australian lime genetics. Front Plant Sci. 2025;16:1614845 10.3389/fpls.2025.1614845 PMC12202233 40584869

[13] Smith MW, Gultzow DL, Reid M. et al. “Extreme hybrids” from the Australian citrus rootstock breeding program. Italus Hortus. 2024;31:43

[14] Nakandala U, Masouleh AK, Smith MW. et al. Haplotype-resolved chromosome-level genome assembly of *Citrus australis* reveals disease resistance and other citrus-specific genes. Hortic Res. 2023b;10:uhad058 10.1093/hr/uhad058 PMC10199705 37213680

[15] Singh K, Huff M, Liu J. et al. Chromosome-scale, *de novo*, phased genome assemblies of three Australian limes. Plants. 2024;13:1460 10.3390/plants13111460 PMC11174732 38891269

[16] Delort E, Yuan YM (2018). Finger lime/The Australian Caviar— Citrus australasica. In: (éditeur), Exotic Fruits. Elsevier, 203–10

[17] Luro F, Bloquel E, Tomu B. et al. The INRA-CIRAD citrus germplasm collection of San Giuliano, Corsica. In: Zech-Matterne V, Fiorentino G (eds.), AGRUMED: Archaeology and History of Citrus Fruit in the Mediterranean. Publications du Centre Jean Bérard: Naples, 2017,

[18] Belser C, Istace B, Denis E. et al. Chromosome-scale assemblies of plant genomes using nanopore long reads and optical maps. Nat Plants. 2018;4:879–87 10.1038/s41477-018-0289-4 30390080

[19] Droc G, Giraud D, Belser C. et al. A super-pangenome for cultivated citrus reveals evolutive features during the allopatric phase of their reticulate evolution. Plant Biotechnol J. 2026;24:3345–67 10.1111/pbi.70553 PMC13110163 41631370

[20] Ollitrault P, Hufnagel B, Curk F. et al. Comparative genetic mapping and a consensus interspecific genetic map reveal strong synteny within the *Citrus* genus. Front Plant Sci. 2024;15:1475965 10.3389/fpls.2024.1475965 PMC11682908 39737380

[21] Haug-Baltzell A, Stephens SA, Davey S. et al. SynMap2 and SynMap3D: web-based whole-genome synteny browsers. Bioinformatics. 2017;33:2197–8 10.1093/bioinformatics/btx144 28334338

[22] D. Giraud *Library of Repeated Sequences Annotated in Citrus Genome Assemblies*. 2025. 10.57745/ZVYEBU, Recherche Data Gouv, V1, UNF:6:rGqbBuEBRBVO4xWBbTJFjA== [fileUNF]

[23] Giraud D, Choisne N, Summo M. et al. Construction of a comprehensive library of repeated sequences for the annotation of *Citrus* genomes. BMC Genom Data. 2025;26:30 10.1186/s12863-025-01321-6 PMC12007355 40247189

[24] Nakandala U, Furtado A, Masouleh AK. et al. The genome of *Citrus australasica* reveals disease resistance and other species-specific genes. BMC Plant Biol. 2024b;24:260 10.1186/s12870-024-04988-8 PMC11005238 38594608

[25] Forster PI, Smith MW. *Citrus wakonai* (Rutaceae), a new species from Papua New Guinea. Austrobaileya. 2010;8:133–8

[26] Wang L, He F, Huang Y. et al. Genome of wild mandarin and domestication history of mandarin. Mol Plant. 2018;11:1024–37 10.1016/j.molp.2018.06.001 29885473

[27] Wu GA, Prochnik S, Jenkins J. et al. Sequencing of diverse mandarin, pummelo and orange genomes reveals complex history of admixture during citrus domestication. Nat Biotechnol. 2014;32:656–62 10.1038/nbt.2906 PMC4113729 24908277

[28] Bayer RJ, Mabberley DJ, Morton C. et al. A molecular phylogeny of the orange subfamily (Rutaceae: Aurantioideae) using nine cpDNA sequences. Am J Bot. 2009;96:668–85 10.3732/ajb.0800341 21628223

[29] Bausher MG, Singh ND, Lee SB. et al. The complete chloroplast genome sequence of *Citrus sinensis* (L.) Osbeck var. ‘Ridge Pineapple’: organization and phylogenetic relationships to other angiosperms. BMC Plant Biol. 2006;6:21 10.1186/1471-2229-6-21 PMC1599732 17010212

[30] Witharana EP, Iwasaki T, San MH. et al. Subfamily evolution analysis using nuclear and chloroplast data from the same reads. Sci Rep. 2025;15:687 10.1038/s41598-024-83292-9 PMC11698846 39753617

[31] Mabberley D . Australian Citreae with notes on other Aurantioideae (Rutaceae). Telopea. 1998;7:333–44

[32] Sallet E, Gouzy J, Schiex T. EuGene: an automated integrative gene finder for eukaryotes and prokaryotes. Methods Mol Biol. 2019;1962:97–120 10.1007/978-1-4939-9173-0_6 31020556

[33] Emms DM, Kelly S. OrthoFinder: phylogenetic orthology inference for comparative genomics. Genome Biol. 2019;20:238 10.1186/s13059-019-1832-y PMC6857279 31727128

[34] Emms DM, Kelly S. OrthoFinder: solving fundamental biases in whole genome comparisons dramatically improves orthogroup inference accuracy. Genome Biol. 2015;16:157 10.1186/s13059-015-0721-2 PMC4531804 26243257

[35] De Bie T, Cristianini N, Demuth JP. et al. CAFE: a computational tool for the study of gene family evolution. Bioinformatics. 2006;22:1269–71 10.1093/bioinformatics/btl097 16543274

[36] Nakandala U, Furtado A, Henry RJ. *Citrus* genomes: past, present and future. Hortic Res. 2025;12:uhaf033 10.1093/hr/uhaf033 PMC11992330 40224327

[37] Berhow MA. et al. Citrus Limonoids: Functional Chemicals in Agriculture and Food. Washington: American Chemical Society; 2000

[38] Ahmed D, Curk F, Evrard JC. et al. Preferential disomic segregation and *C. micrantha/C. medica* interspecific recombination in tetraploid ‘Giant Key’ lime: outlook for triploid lime breeding. Front Plant Sci. 2020;11:939 10.3389/fpls.2020.00939 PMC7330052 32670332

[39] Calvez L, Dereeper A, Mournet P. et al. Intermediate inheritance with disomic tendency in tetraploid intergeneric *Citrus × Poncirus* hybrids enhances the efficiency of citrus rootstock breeding. Agronomy. 2020;10:1961

[40] Bruyère S, Luro F, Froelicher Y. et al.. Abstract book Sustainable citriculture: the role of applied knowledge. In: Dirceu M, Eduardo FC, Valdenice MN. et al. (eds.), *International Citrus Congress, Foz do Iguaçu (Brazil)*. Londrina: IAPAR, 2016, 125.

[41] Ollitrault P, Curk F, Ollitrault F. et al. Usefulness of phylogenetic diagnostic SNP markers of citrus ancestral taxa for genetics and breeding. In: Dirceu M, Eduardo FC, Valdenice MN. et al. (eds.), Proceedings of the International Citrus Congress. 2016. Abstract book Sustainable citriculture: the role of applied knowledge. Londrina: IAPAR, 126–7

[42] Oueslati A, Salhi-Hannachi A, Luro F. et al. Genotyping by sequencing reveals the interspecific admixture along the genomes of modern citrus varieties. PLoS One. 2017;12:e0185618 10.1371/journal.pone.0185618 PMC5628881 28982157

[43] Ahmed D, Comte A, Curk F. et al. Genotyping by sequencing can reveal the complex mosaic genomes in gene pools resulting from reticulate evolution: a case study in diploid and polyploid citrus. Ann Bot. 2019;123:1231–51 10.1093/aob/mcz029 PMC6612944 30924905

[44] Wu GA, Sugimoto C, Kinjo H. et al. Diversification of mandarin citrus by hybrid speciation and apomixis. Nat Commun. 2021;12:4377 10.1038/s41467-021-24653-0 PMC8313541 34312382

[45] Calvez L, Dereeper A, Perdereau A. et al. Meiotic behaviors of allotetraploid citrus drive the interspecific recombination landscape, the genetic structures, and trait inheritance in tetrazyg progenies aiming to select new rootstocks. Plants. 2023;12:1630 10.3390/plants12081630 PMC10146282 37111854

[46] Chen S, Zhou Y, Chen Y. et al. fastp: an ultra-fast all-in-one FASTQ preprocessor. Bioinformatics. 2018;34:i884–90 10.1093/bioinformatics/bty560 PMC6129281 30423086

[47] Goel M, Sun H, Jiao WB. et al. SyRI: finding genomic rearrangements and local sequence differences from whole-genome assemblies. Genome Biol. 2019;20:277 10.1186/s13059-019-1911-0 PMC6913012 31842948

[48] Marçais G, Delcher AL, Phillippy AM. et al. MUMmer4: a fast and versatile genome alignment system. PLoS Comput Biol. 2018;14:e1005944 10.1371/journal.pcbi.1005944 PMC5802927 29373581

[49] Li L, Stoeckert CJ, Roos DS. OrthoMCL: identification of ortholog groups for eukaryotic genomes. Genome Res. 2003;13:2178–89 10.1101/gr.1224503 PMC403725 12952885

[50] Sun J, Lu F, Luo Y. et al. OrthoVenn3: an integrated platform for exploring and visualizing orthologous data across genomes. Nucleic Acids Res. 2023;51:W397–403 10.1093/nar/gkad313 PMC10320085 37114999

[51] Minh BQ, Schmidt HA, Chernomor O. et al. IQ-TREE 2: new models and efficient methods for phylogenetic inference in the genomic era. Mol Biol Evol. 2020;37:1530–4 10.1093/molbev/msaa015 PMC7182206 32011700

