# Supplementary material for: Chromosome-scale genome assembly of Australian finger lime and resequencing reveal the hidden diversity of Oceanian citrus
Source: Hortic Res. 2026 Jun 2;13(11):uhag213. doi: 10.1093/hr/uhag213 (PMC13719177; doi:10.1093/hr/uhag213)
Supplement: Supplementary file 1 [file web_material_uhag213.zip › Supplementary Figures_Hufnagel_etal_vf_review.pdf]

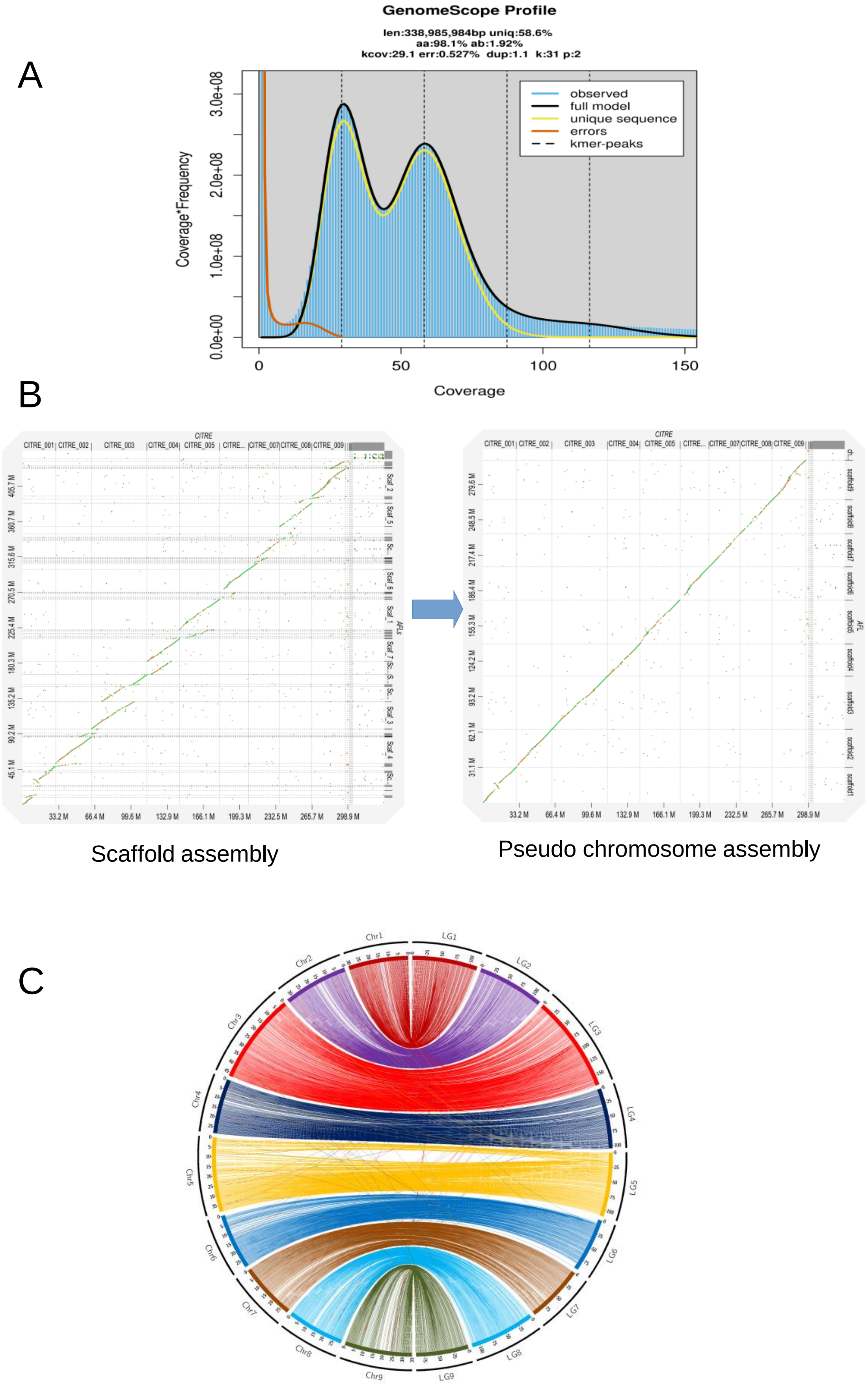


**Supplementary Figure 1: Interspecific mosaic structure of the sequenced accession and implication in duplicated areas on the genome assembly**. **A)** GenomeScope2.0 k-mer analysis based on Illumina short reads estimates a genome size of 338 Mb and a heterozygosity rate of 1.92%, supporting the hybrid nature of this accession. **B)** DotPlot of preliminary scaffold assembly (left) and final pseudochromosome scale assembly with the CITRE genome (Cleopatra mandarin; Droc *et al.* 2024). **C)** Anchorage of 7819 genes the *Citrus* consensus genetic map (Ollitrault *et al.* 2024) on the pseudochromosome assembly.

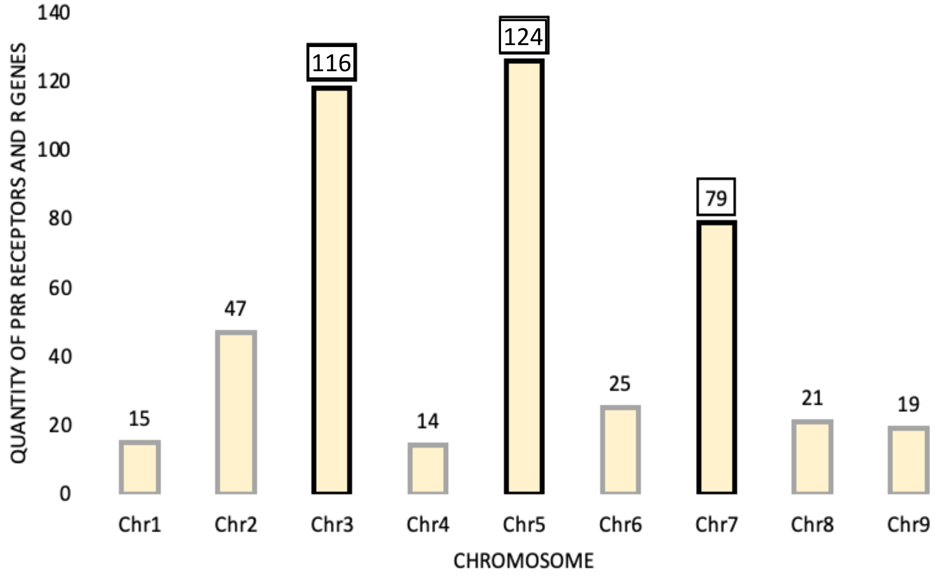


**Supplementary Figure 2:** Distribution of PRR receptors and R genes from the well-defined classes across the chromosomes of AFL SRA 899.

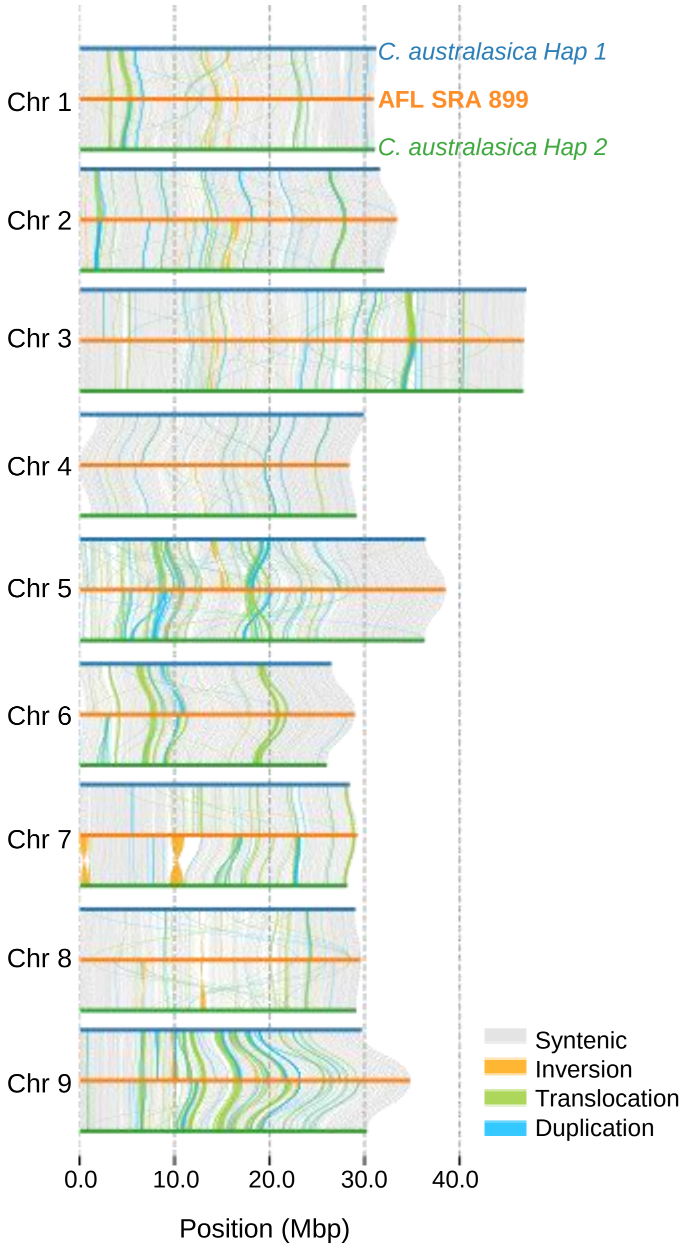


**Supplementary Figure 3:** Comparative structural genomics between AFL SRR 899 and the two haplotypes of *Citrus australasica* assemblies as reported by Nakandala *et al*. (2024). Conserved syntenic blocks are depicted in grey, while regions lacking alignment are shown in white. Distinct categories of genomic rearrangements are color-coded according to their type. Structural variation was assessed using the Synteny and Rearrangement Identifier (SyRI) tool.

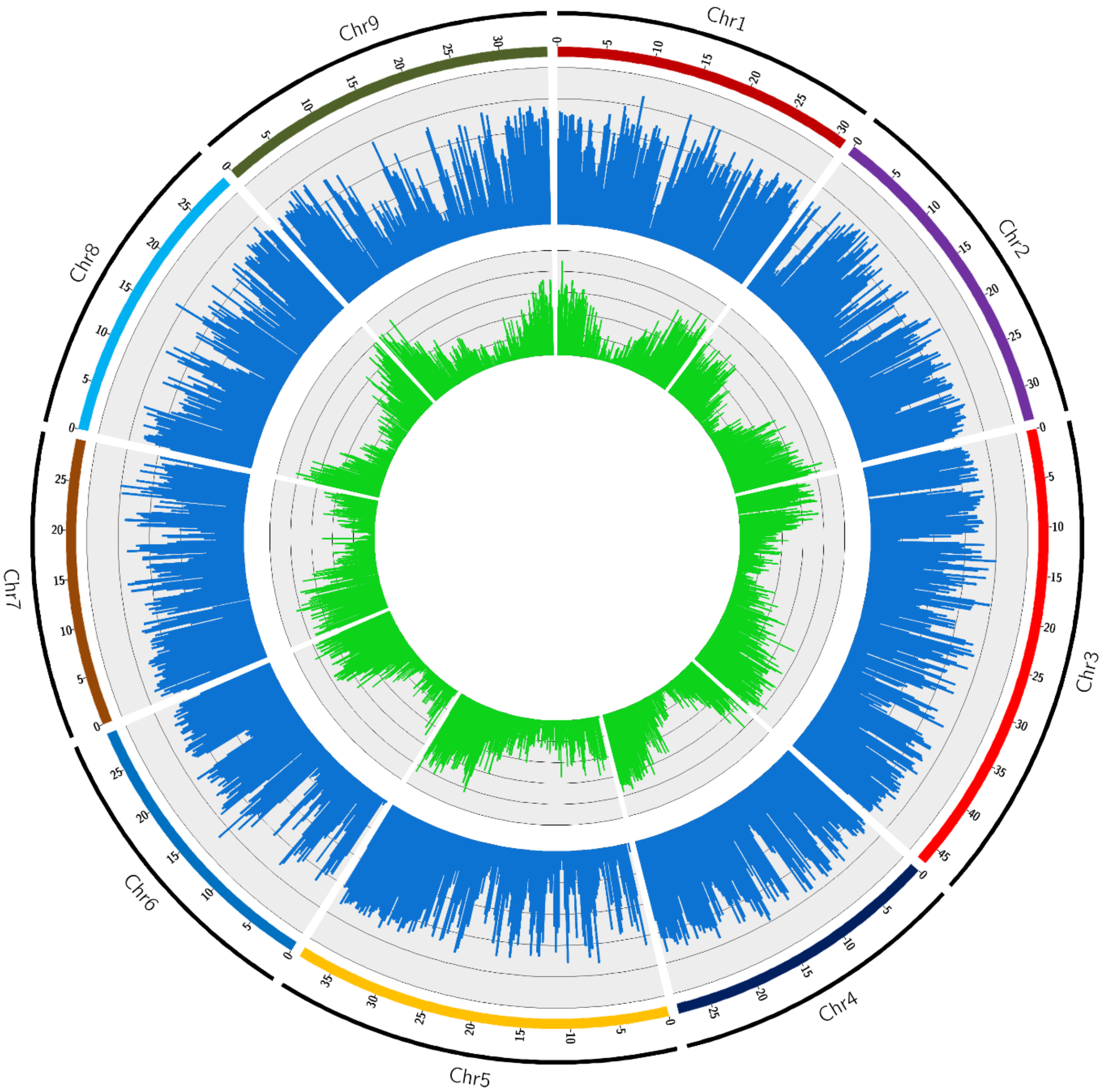


**Supplementary Figure 4:** Distribution of SNPs and genes along the AFL genome. External ring: SNP density; Windows of 200 kb; scale 0 to 50000 SNPs / 200 kb. Internal ring: Gene density Windows of 200 kb; scale 0 to 50 genes / 200 kb.

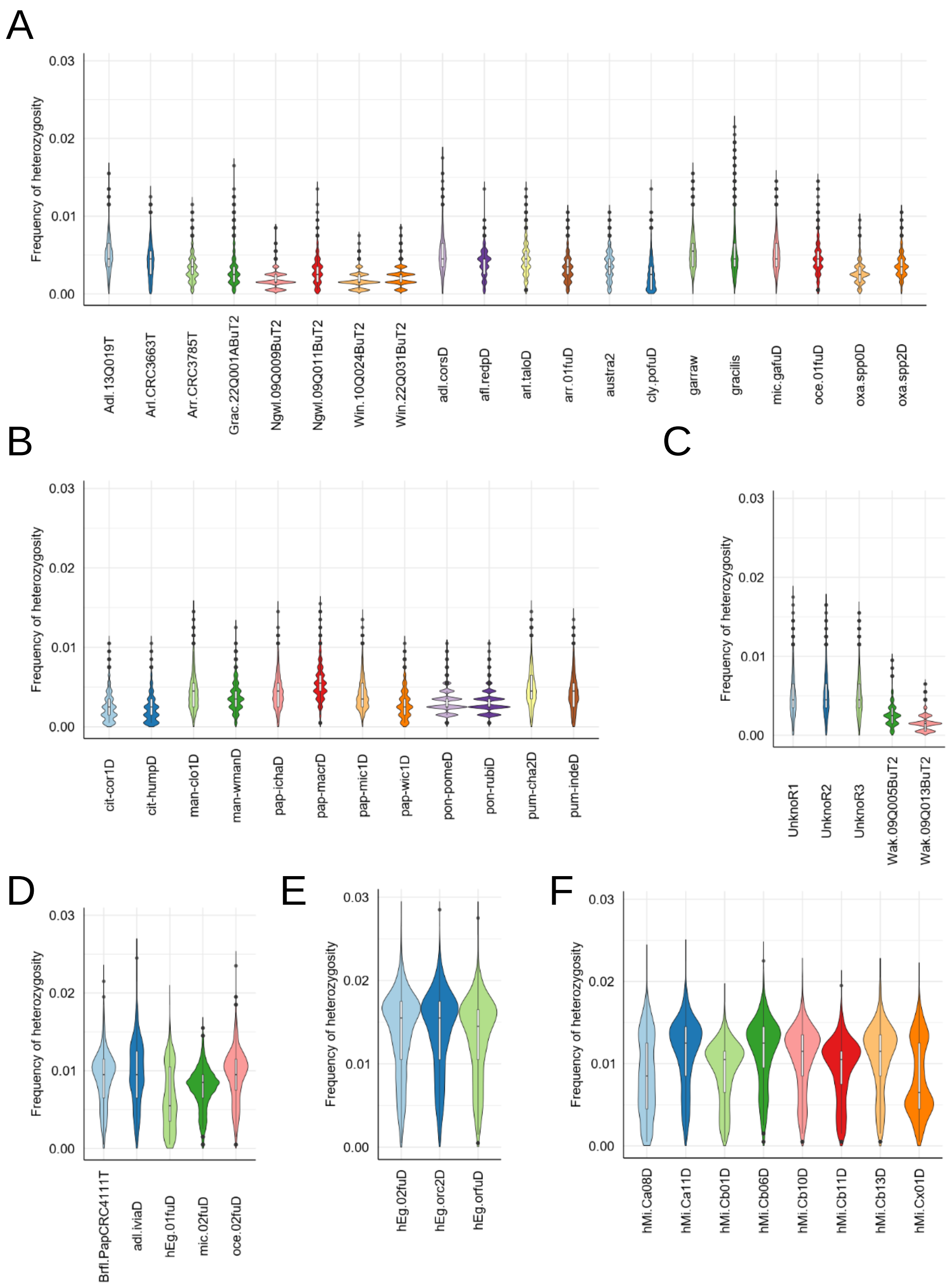


**Supplementary Figure 5: Heterozygosity value distribution (frequency of heterozygosity in successive 200 kb windows)**. **A)** Oceanian species representatives; **B)** Asian species representatives; **C)** Unknown Cape York sp. and *C. wakonai* representatives; **D)** Other interspecific Oceanian accessions; **E)** Eremorange accessions; F) Randomly admixed hybrids of the Cirad breeding project.

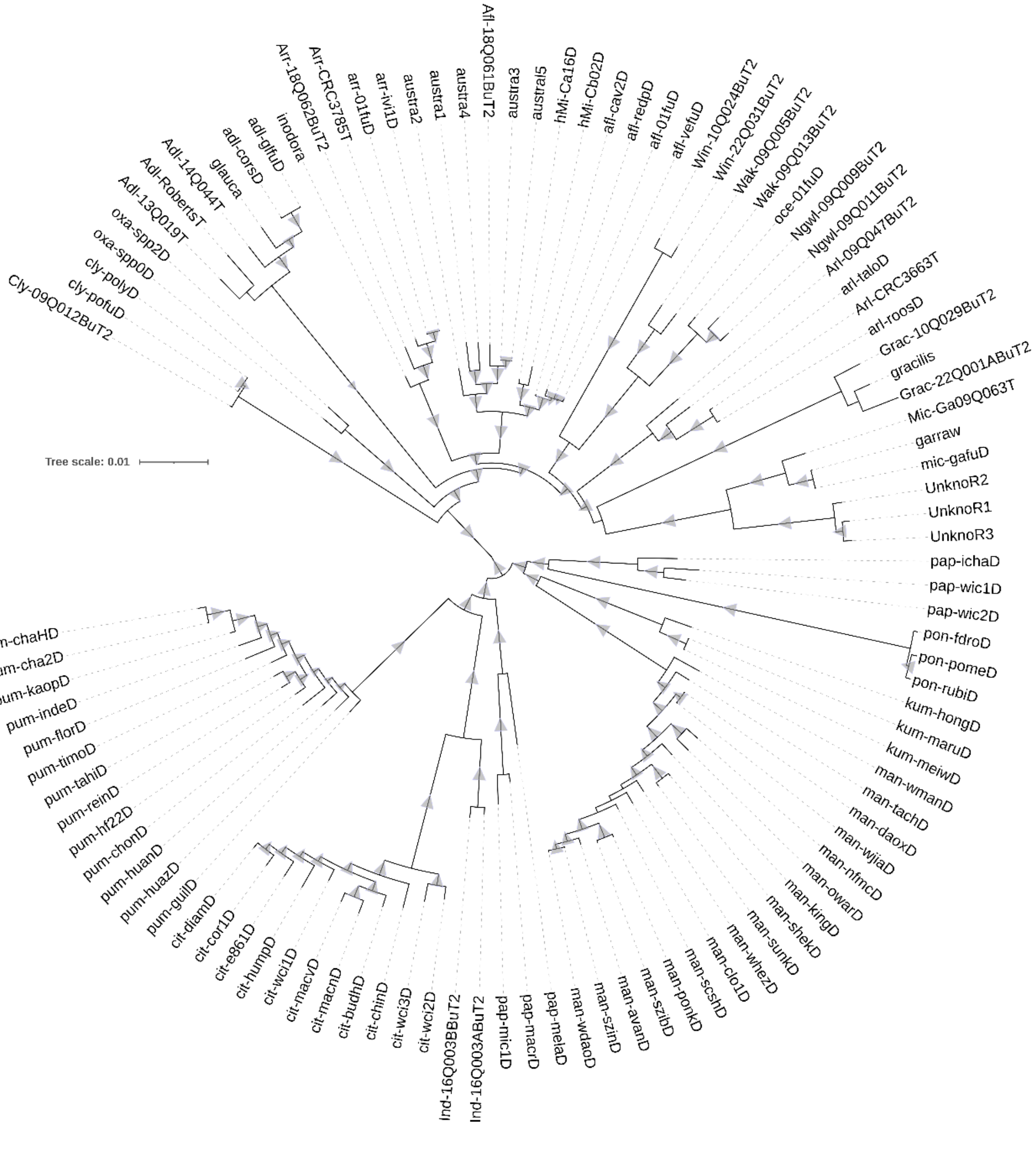


**Supplementary Figure 6:** Maximum likelihood phylogenetic tree based on 1,322,933 nuclear SNPs, with explicated codes for each genotype. Triangles indicate branch support (Boostrap >90; 1000 replicates), with size proportional to support values. The scale bar represents 0.01 substitutions per site. Genotypes list is available in Supplementary Table 5.

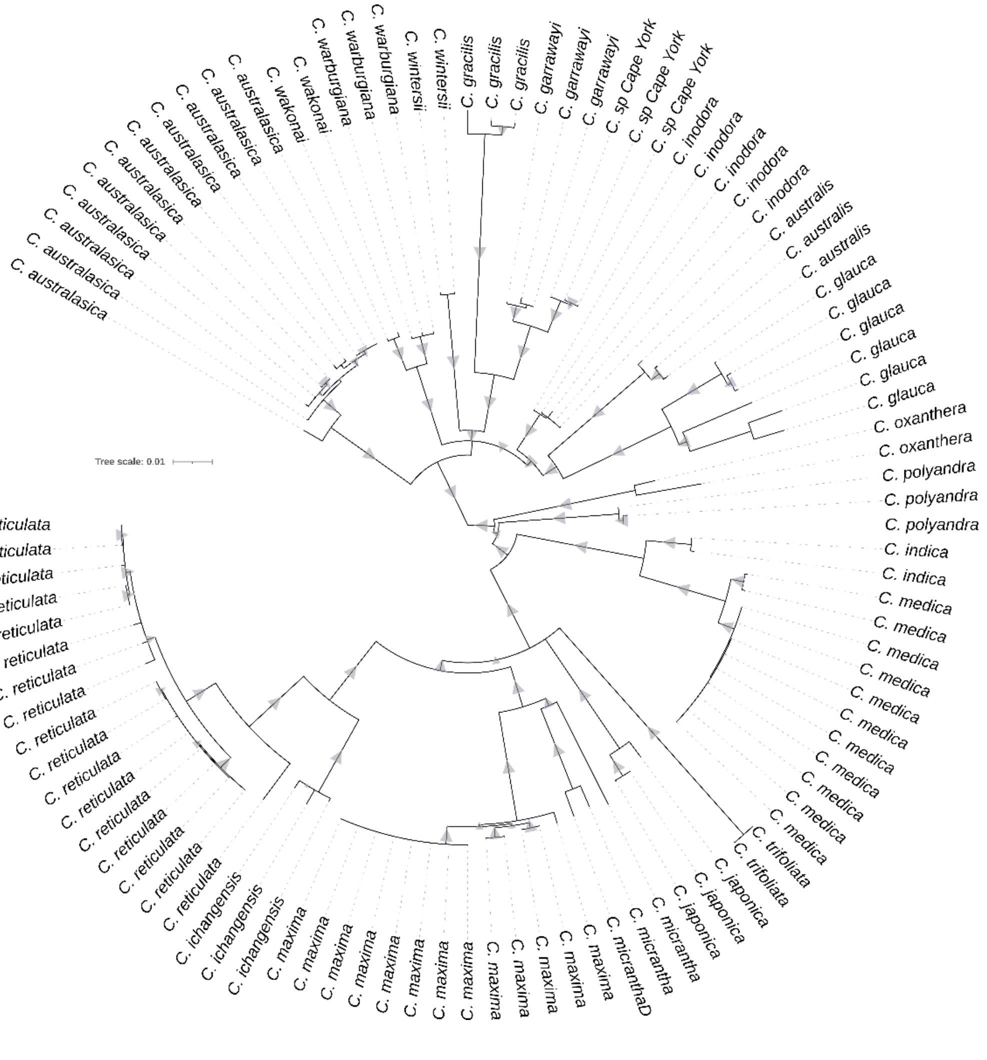


**Supplementary Figure 7:** Maximum likelihood phylogenetic tree based on 3,695 SNPs derived from the *C. sinensis* plastome, Triangles indicate branch support (Boostrap >90; 1000 replicates), with size proportional to support values. The scale bar represents 0.01 substitutions per site.

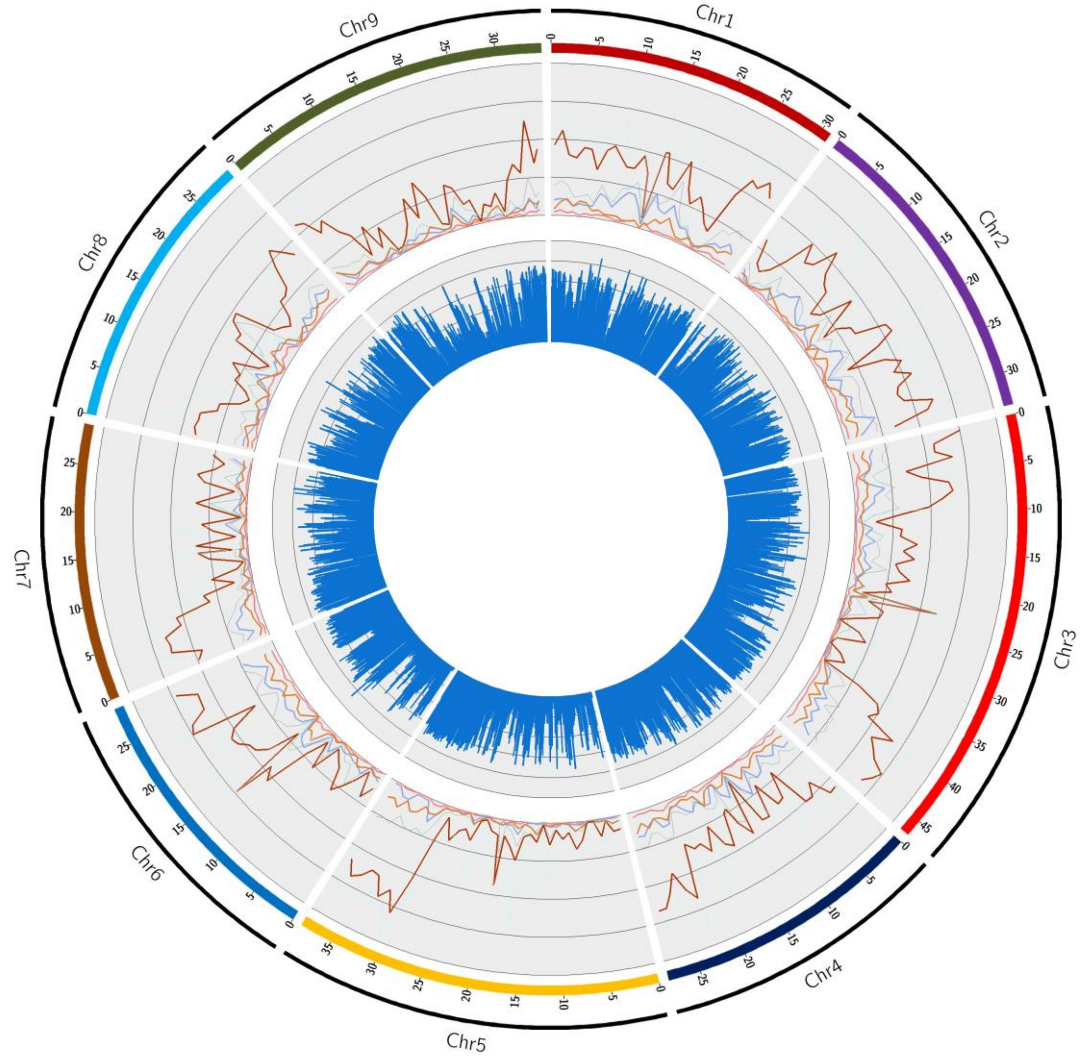


**Supplementary Figure 8:** Distribution along the genome of the DSNPs of Australian species. Internal ring: SNP density. Windows of 200 kb; scale 0 to 50000 SNPs / 200 kb. External ring: DSNP density; dark brown: *C. glauca*; light brown: *C. inodora*; salmon: *C. australasica*, light blue: *C. australis*; peacock blue: *C. garrawayi*; + Unknown Cape York sp.; sky-blue: *C. gracilis*. Windows of 1Mb; scale: 0 to 4000 DSNPs / Mb.

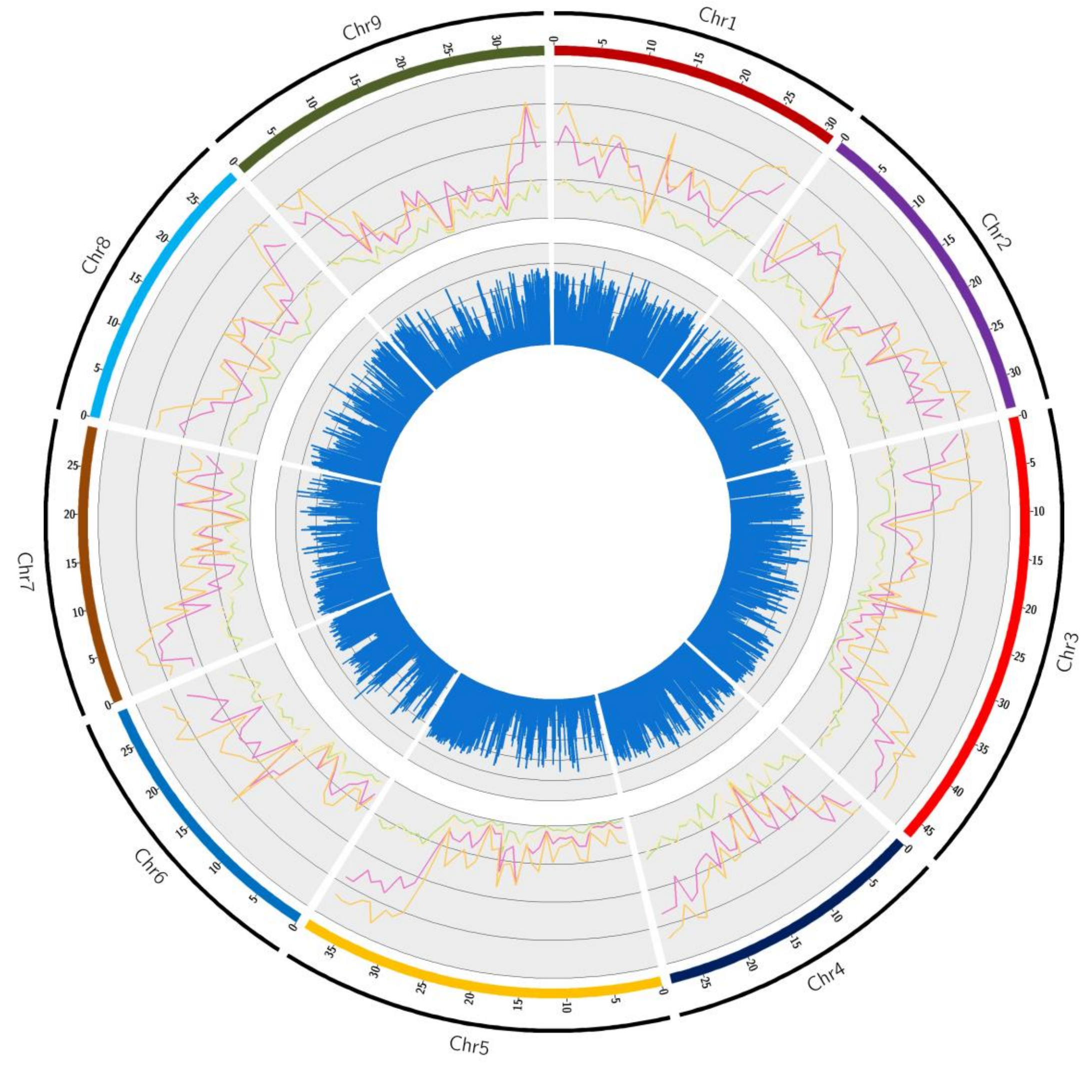


**Supplementary Figure 9:** Distribution along the genome of the DSNPs of Papua-New-Guinean and New-Caledonian species. Internal ring: SNP density. Windows of 200 kb; scale 0 to 50000 SNPs / 200 kb. External ring: DSNP density; pink: *C. wintersii*; yellow-green: *C. warburgiana* + *C. wakonai*; sunset yellow: *C. polyandra*; blond: *Oxanthera* sp. Windows of 1Mb; scale: 0 to 4000 DSNPs / Mb.

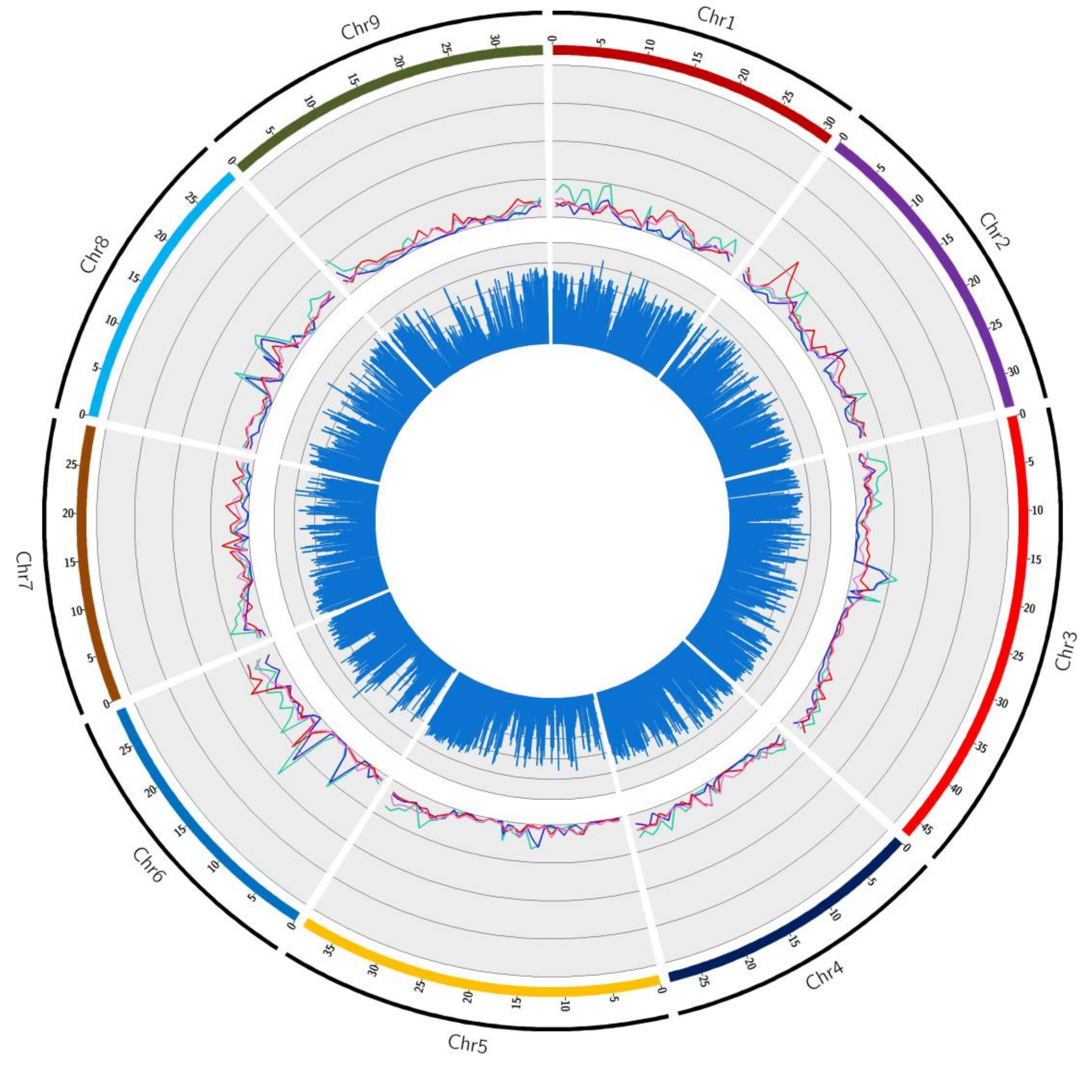


**Supplementary Figure 10**: Distribution along the genome of Oceanian DSNPs of the Oceanian subclades. Internal ring: SNP density. Windows of 200 kb; scale 0 to 50000 SNPs / 200 kb. External ring: DSNP density; green: Unknown Cape York sp.; blue: *C. garrawayi*; red: *C. warburgiana*; pink: *C. wakonai*. Windows of 1Mb; scale: 0 to 4000 DSNPs / Mb.

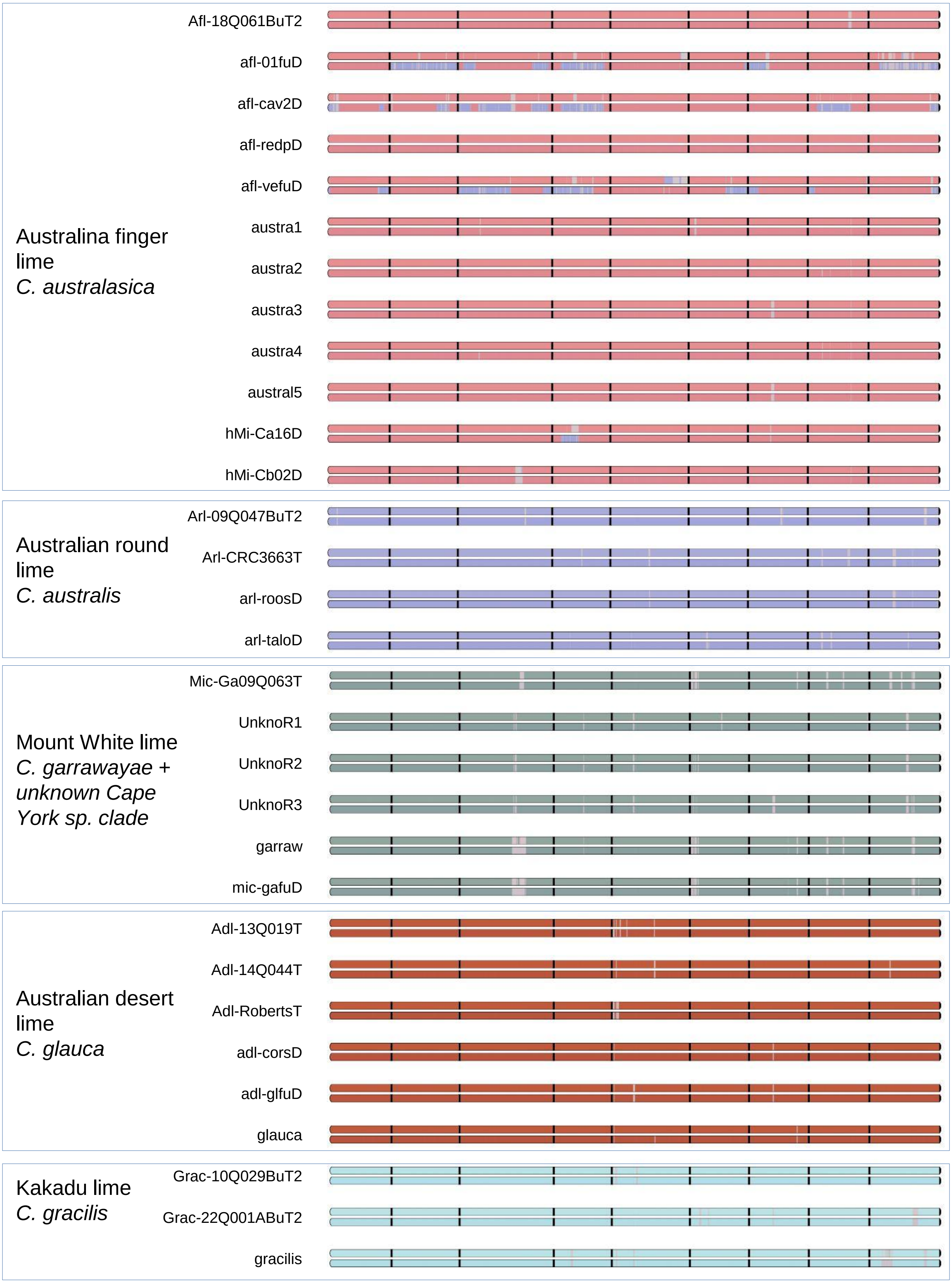


**Supplementary figure 11:** Phylogenomic mosaic structure along the nine chromosome (separated by black dash) of the accessions representatives of the main Oceanian clades (first part).

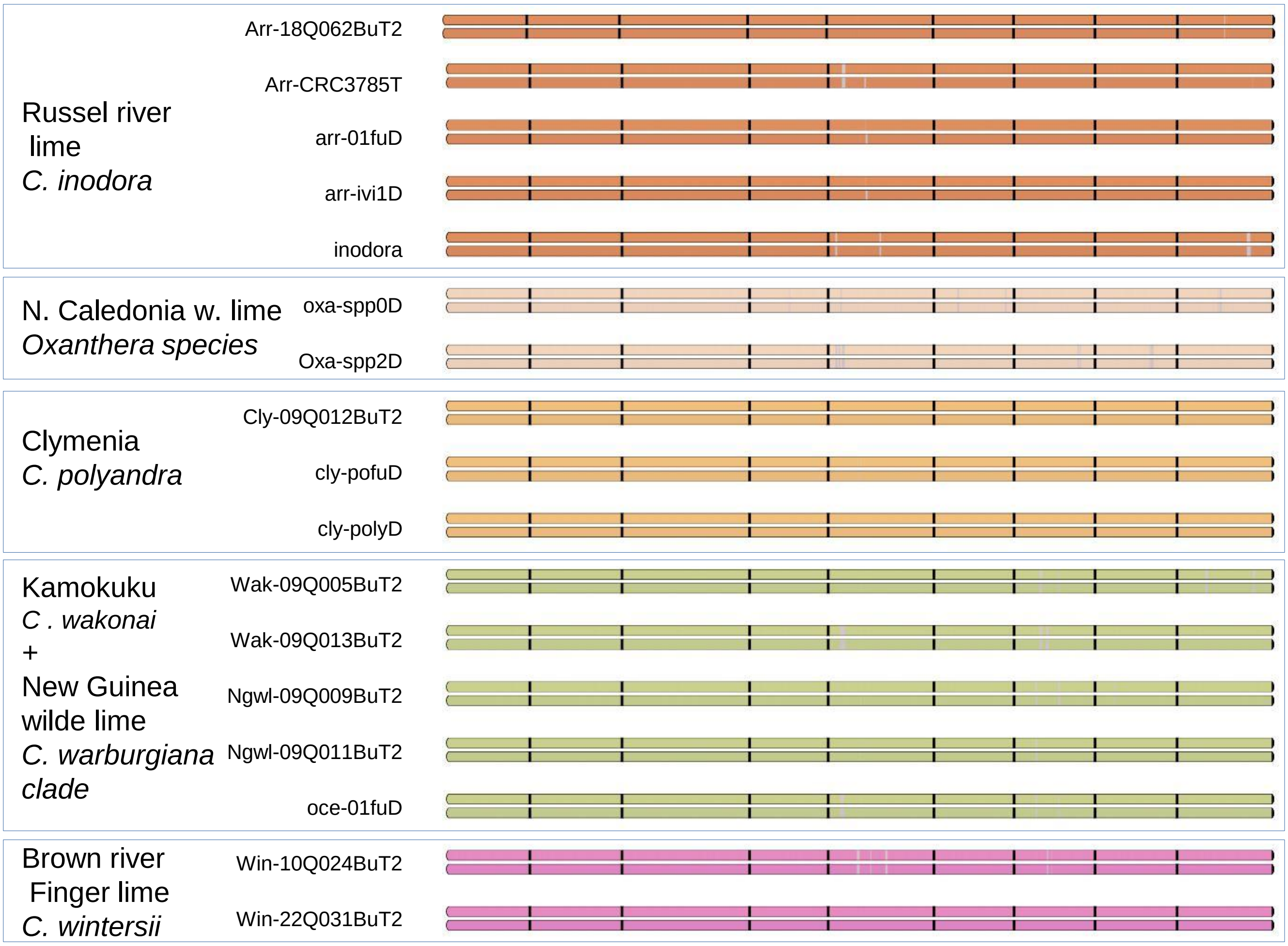


**Supplementary figure 11 (cont.):** Phylogenomic mosaic structure along the nine chromosome (separated by black dash) of the accessions representatives of the main Oceanian clades (second part).

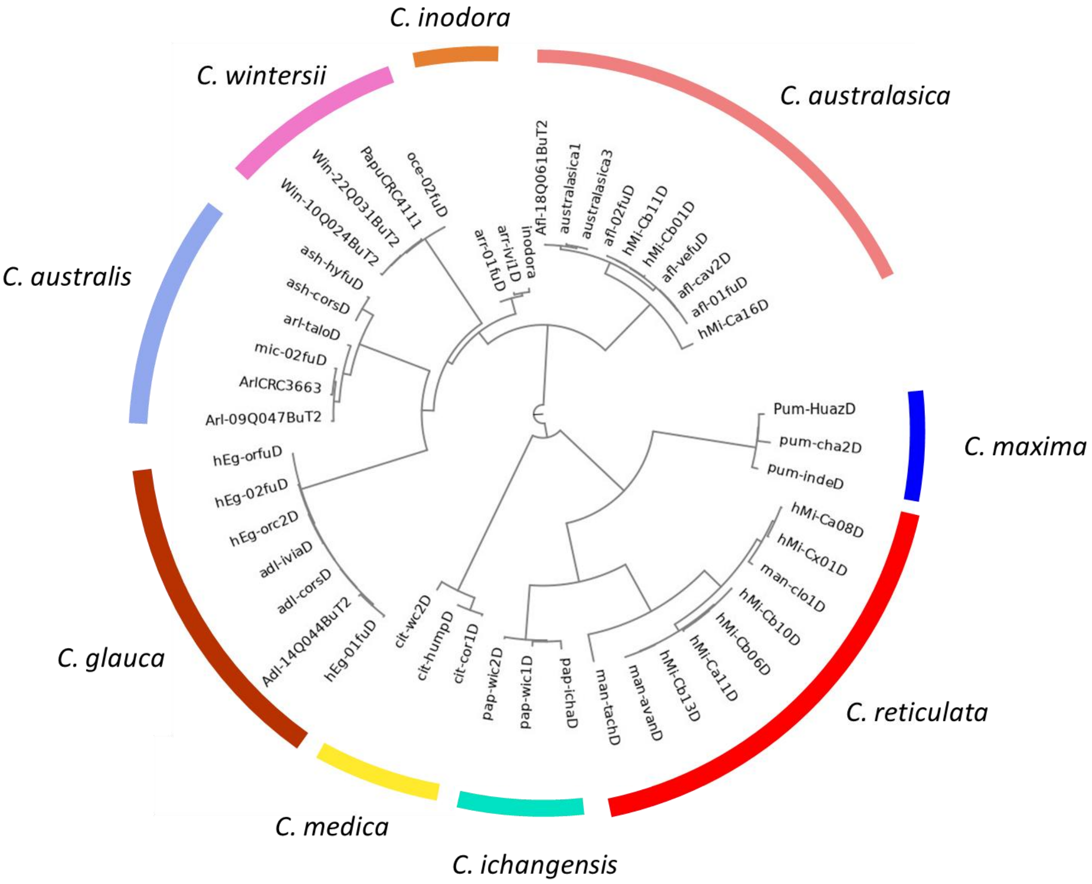


**Supplementary Figure 12:** Chloroplast inheritance of the analysed Oceanian admixed accessions

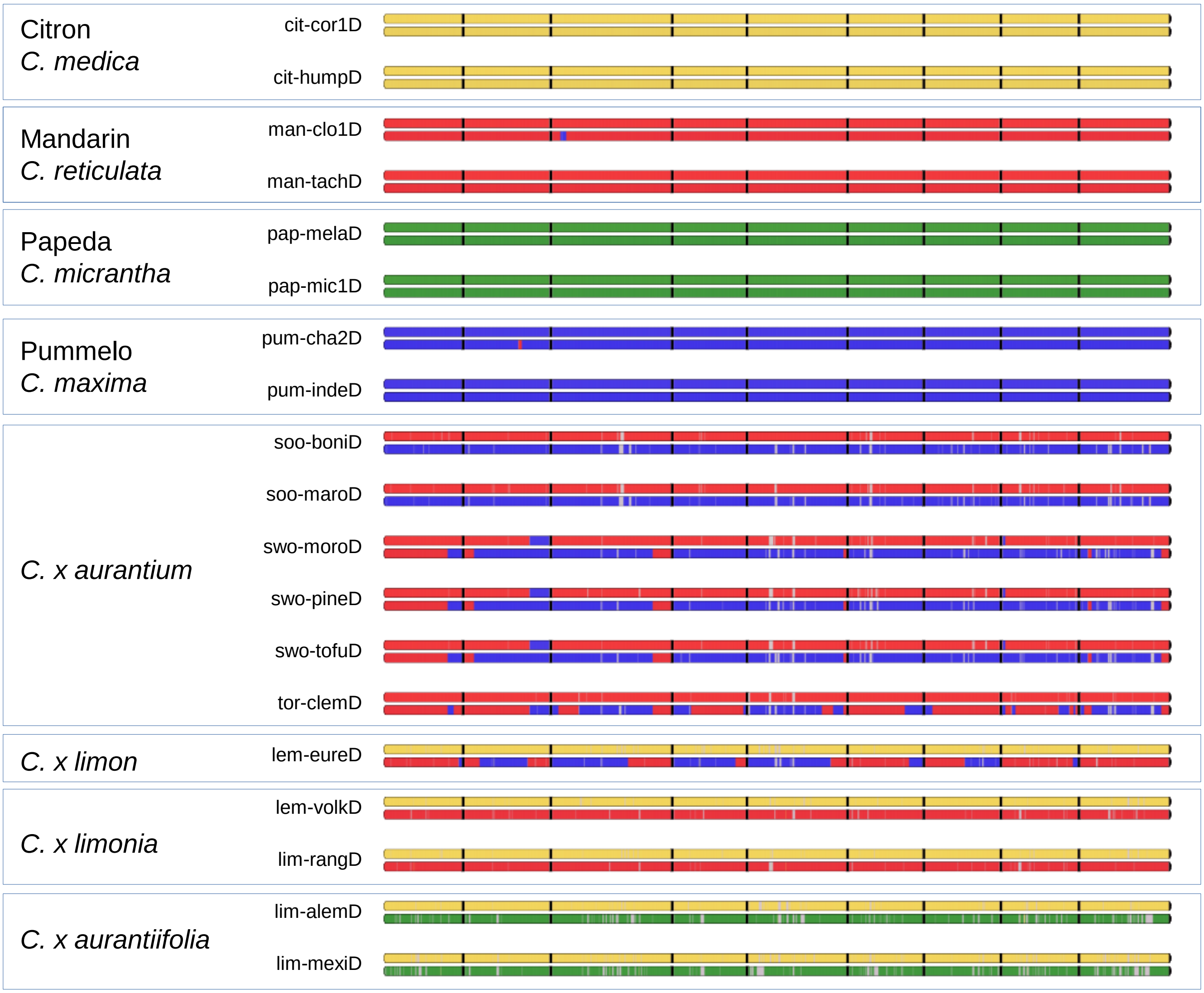


**Supplementary Figure 13:** Examples of Phylogenomic mosaic structure along the nine chromosome (separated by black dash) of some accessions representatives of the main Asian clades and admixed horticultural groups.

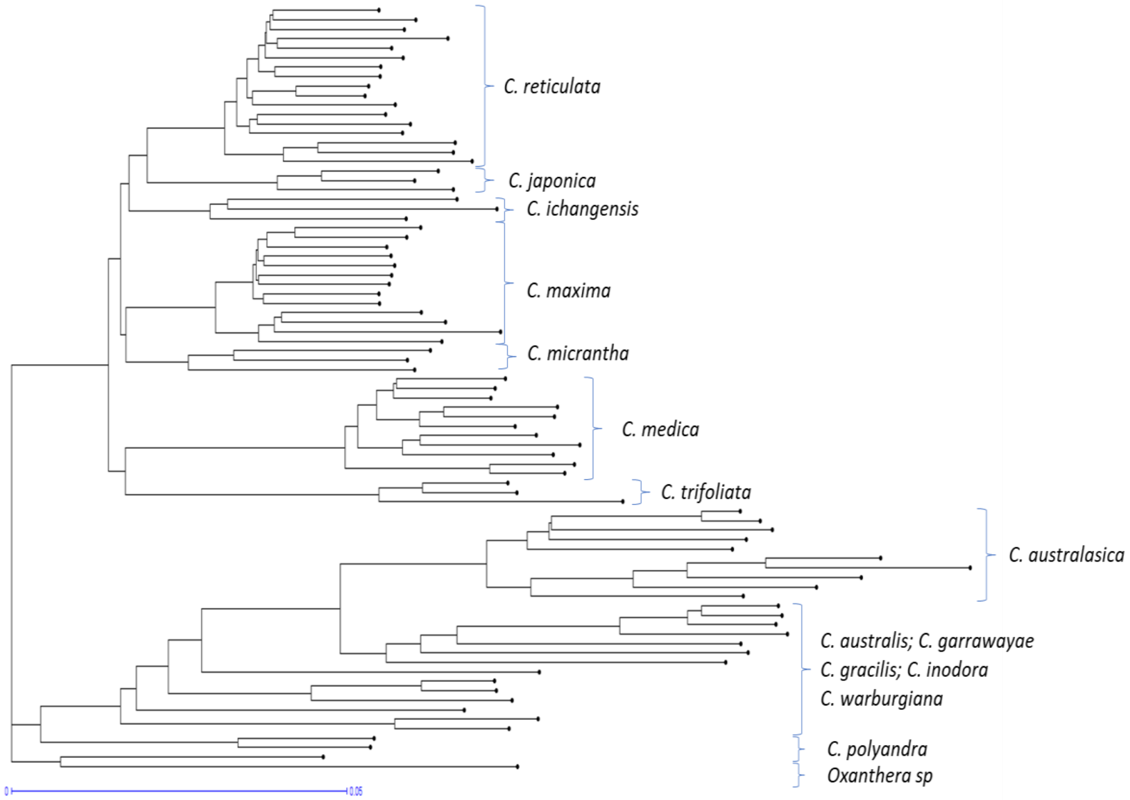


**Supplementary Figure 14. Neighbor-Joining tree based on gene presence/absence variation (PAV) across 81 citrus accessions.** The tree was constructed from binary gene loss data using SGSGeneLoss outputs mapped to the *AFL SRA 899* reference genome.

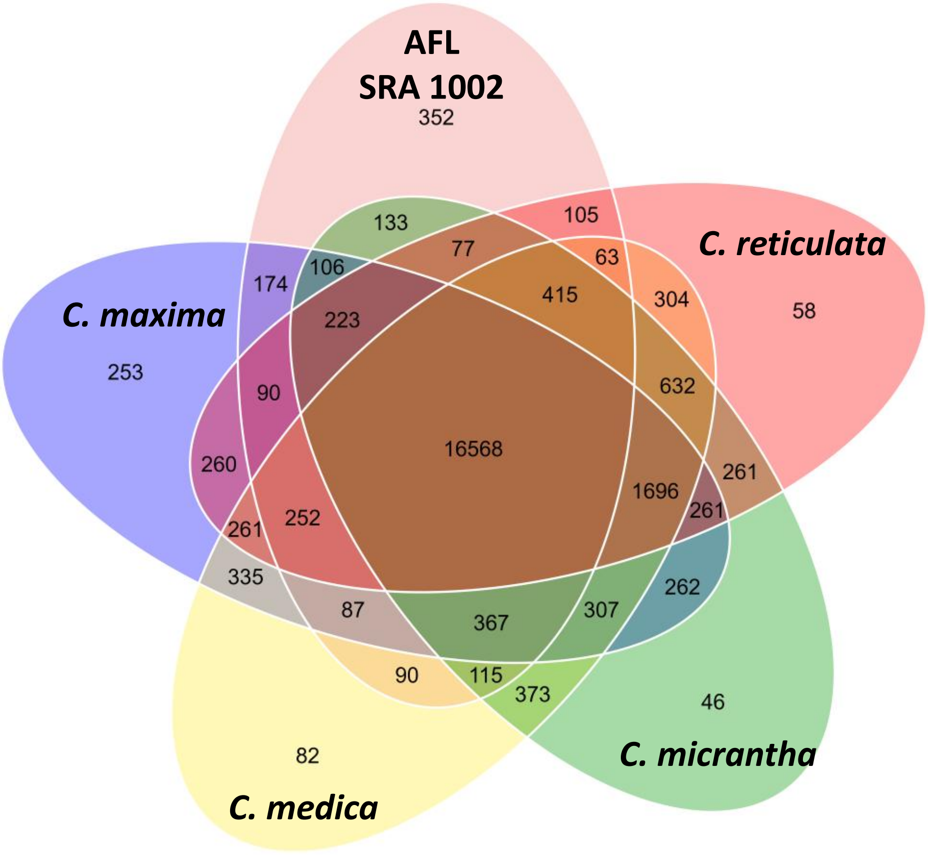


**Supplementary Figure 15. Distribution of orthogroups across five citrus genomes.** Venn diagram showing the number of shared and species-specific orthogroups among AFL SRA 1002, *Citrus reticulata*, *C. maxima*, *C. medica*, and *C. micrantha*. The majority of orthogroups (16,568) are conserved across all five genomes, forming a large core gene set, while smaller subsets correspond to lineage-specific and partially shared gene families.

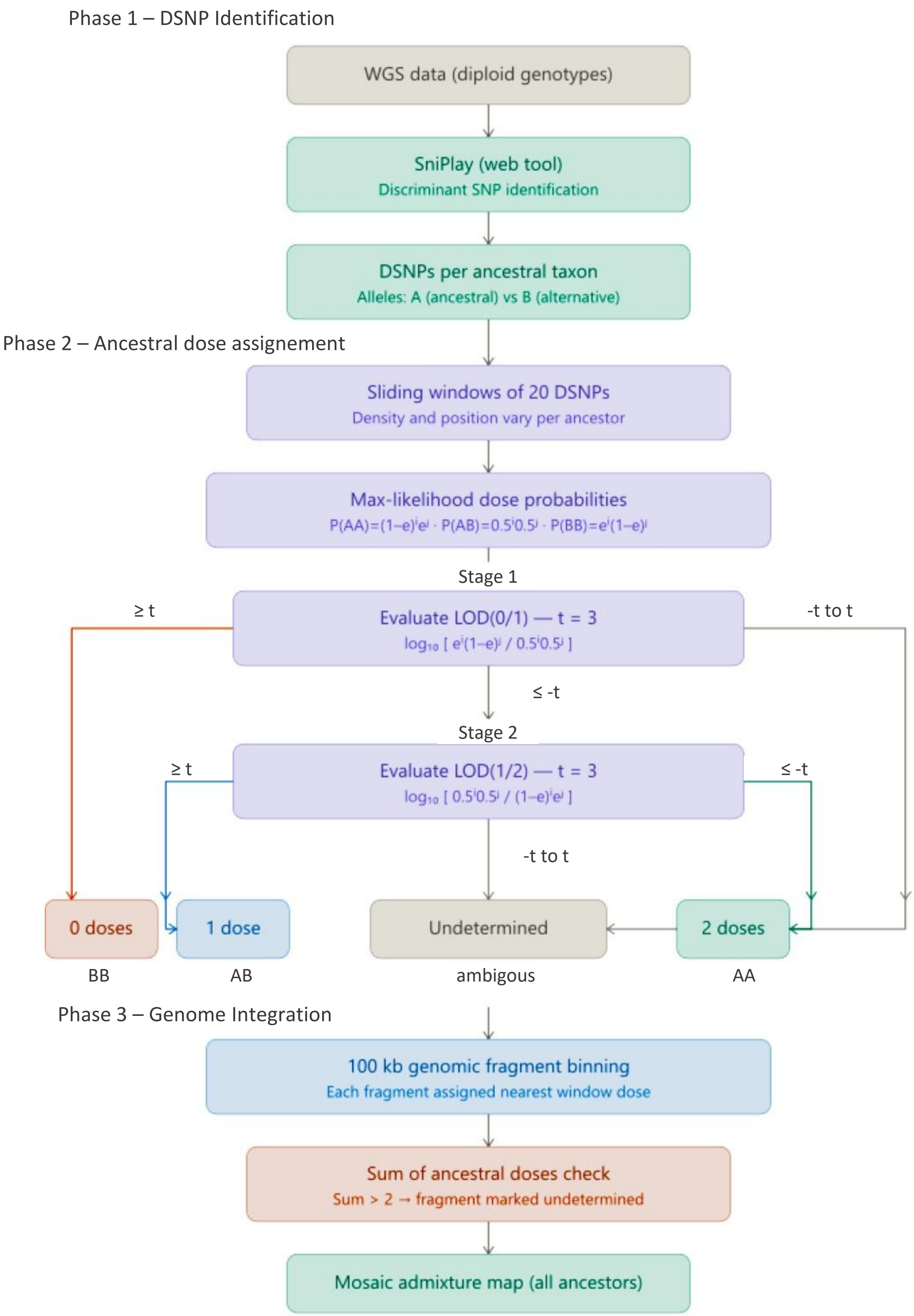


**Supplementary Figure 16. Pipeline for DSNP identification and ancestral admixture analysis.** Phase 1 (green): DSNPs are identified per ancestral taxon using SniPlay from diploid WGS genotypes. Phase 2 (purple): ancestral doses are assigned within sliding windows of 20 DSNPs via a two-stage LOD score decision rule (threshold $t = 3$), yielding five possible outcomes per window — 0 doses (BB), 1 dose (AB), 2 doses (AA), or undetermined at either stage. Phase 3 (blue): the genome is binned into 100 kb fragments, each assigned the dose of its overlapping window; fragments where the sum of ancestral contributions exceeds 2 are marked undetermined. The final output is a genome-wide mosaic admixture map. See Supplementary Note 2 for full details of the statistical framework.
