# Supplementary material for: Chromosome-scale genome assembly of Australian finger lime and resequencing reveal the hidden diversity of Oceanian citrus
Source: Hortic Res. 2026 Jun 2;13(11):uhag213. doi: 10.1093/hr/uhag213 (PMC13719177; doi:10.1093/hr/uhag213)
Supplement: Supplementary file 1 [file web_material_uhag213.zip › Supplementary Notes_Hufnagel et al_R1.pdf]

Supplementary Note 1: *De novo* AFL 1002 genome assembly and annotation

De novo nuclear genome assembly

The *de novo* genome assembly was obtained by combining nanopore long-read sequencing, Illumina PCR-Free sequencing and bionano optical mapping. The final pseudochromosome assembly was guided by Dotplot with *C. reticulata* CITRE genome assembly (Droc *et al.* 2024) and integration with an Oceanian citrus hybrid genetic map.

PromethION library preparation and sequencing

The libraries were prepared according to the '1D Genomic DNA by ligation (Kit 9 chemistry)' protocol, provided by Oxford Nanopore. Genomic DNA was first repaired and end-prepped with the NEBNext FFPE Repair Mix (New England Biolabs, Ipswich, MA, USA) and the NEBNext® Ultra™ II End Repair/dA-Tailing Module (NEB). DNA was then purified with AMPure XP beads (Beckmann Coulter, Brea, CA, USA), and sequencing adapters provided by Oxford Nanopore Technologies were ligated using Concentrated T4 DNA Ligase 2M U/ml (NEB). After purification with AMPure XP beads (Beckman Coulter) using dilution buffer (ONT) and wash buffer (ONT), the library was mixed with the sequencing buffer (ONT) and the Library Loading Bead (ONT) and loaded on the PromethION R9.4.1 Flow Cells. The nanopore long reads were not cleaned, and raw reads were used for genome assembly.

Illumina PCR-Free library preparation and sequencing

PCR free libraries were prepared using the Kapa Hyper Prep Kit (Roche, Basel, Switzerland). Briefly, genomic DNA (1-1.5µg) was sonicated using a Covaris E220 sonicator (Covaris, Woburn, MA, USA). Fragments were end-repaired and 3'-adenylated, and Illumina adapters (Bioo Scientific, Austin, TX, USA) were ligated following the manufacturer's instructions. Ligation products were purified twice with AMPure XP beads (Beckman Coulter Genomics,

Danvers, MA, USA) and quantified by qPCR (MxPro, Agilent Technologies, Santa Clara, CA, USA) using the KAPA Library Quantification Kit for Illumina Libraries (Roche). The library profile was assessed using an Agilent High Sensitivity DNA kit on the Agilent 2100 Bioanalyser. Libraries were paired-end sequenced on an Illumina HiSeq2500 instrument (Illumina, San Diego, CA, USA) using 250 base-length read chemistry. After the Illumina sequencing, an in-house quality control process was applied to the reads that passed the Illumina quality filters. The first step discards low-quality nucleotides (Q < 20) from both ends of the reads. Next, Illumina sequencing adapters and primer sequences were removed from the reads. Then, reads shorter than 30 nucleotides after trimming were discarded. These trimming and removal steps were completed using in-house-designed software based on the FastX package (Engelen and Aury; https://www.genoscope.cns.fr/fastxtend). The last step is to identify and discard read pairs that are mapped to the phage phiX genome, using SOAP aligner (Li *et al.* 2009) , and the Enterobacteria phage PhiX174 reference sequence (GenBank: NC_001422.1). This processing, described (Alberti *et al.* 2018), resulted in high-quality data.

Optical mapping

The direct label and stain labelling (using the DLE-1 enzyme) and the nick label repair and stain (NLRS) labelling (using the BspQI enzyme) protocols and Bionano Genomics protocols were followed with 750ng and 600ng of DNA, respectively. The chip loadings were performed as Bionano Genomics recommended.

Long-read-based genome assembly

First, we estimated genome size and heterozygosity rate using Genomescope2.0 (Ranallo-Benavidez, Jaron and Schatz 2020) with the Illumina reads as inputs. Using these values, we launched the Necat assembler (Chen *et al.* 2021) with the appropriate genome size and with all

Nanopore reads as inputs. Each assembly was then polished two times with HAPO-G (Aury and Istace 2021) and Illumina reads.

Integration of the AFL genome assembly and Oceanian citrus genetic map

Previously published GBS data of 171 'Fortune' mandarin × (*C. australis* × *C. inodora*) hybrids (Ollitrault *et al.* 2024) were used for variant calling using the new AFL genome assembly in pseudochromosomes as template. Detailed information for GBS analysis using ApeK I (Elshire *et al.* 2011) variant calling with the VCF-Hunter 2.1.0 pipeline (https://github.com/SouthGreenPlatform/VcfHunter; (Baurens *et al.* 2019), and genetic mapping with JoinMap5 (https://www.kyazma.nl/index.php/JoinMap/) can be found in Ollitrault *et al.* (2024).

Scaffold sequence reconstruction

Hybrid scaffolding: the BspQI and DLE-1 preparations were run on a single flow cell each. The generated molecules were assembled to produce optical maps using software provided by Bionano Genomics with the following two options: 'add pre-assembly' and non-haplotype without extend and split' (bionano solve and tools Version: 3.3_10252018). The two optical maps and the ONT contigs were subjected to the 2-enzyme hybrid scaffolding pipeline to generate the hybrid scaffolds. BisCoT (Istace, Belser and Aury 2020) was used to correct artifactual duplications (negative gaps) introduced during the scaffolding process.

Dotplot analysis

Comparisons between genome assemblies was performed using dotplot analysis with D-Genies (https://dgenies.toulouse.inra.fr/) with the Minimap2 v2.28 and "many repeats" options.

Pseudochromosome construction

The final genome assembly in pseudochromosomes was guided by dotplot analysis using the *C. reticulata* CITRE genome assembly in pseudochromosome as template (Droc *et al.* 2024) and the integration of the genetic map of the interspecific Oceanian hybrid *C. australis* × *C. australasica*. Chromosome numbering and orientation are identical to those of *C. clementina* V1.0 (https://phytozome-next.jgi.doe.gov/info/Cclementina_v1_0) and P. trifoliata V1.3.1 (https://phytozome-next.jgi.doe.gov/info/Ptrifoliata_v1_3_1).

Annotation of the Australian finger lime assembly

RNA-Seq experiment and transcriptome assembly

Three replicates of young leaves, mature leaves, flowers and fruit pulp from the Australian finger lime SRA 1002 were harvested on adult trees of the Corsican field collection hosted by the Citrus Biological Resource Center (Citrus BCR (INRAE-Cirad, San Giuliano, Corsica, France, Luro *et al.* 2017). RNA was extracted using the NucleoMag® kit (Macherey-Nagel GmbH & Co.KU, Düren, Germany) with the Kingfisher automated system (KingFisher Flex Purification System, ThermoFisher Scientific, Waltham, MA) according to the manufacturer's protocol. RNA integrity and quality were checked on TapeStation, screentape D5000. RNAseq libraries were prepared to obtain labelled and matched sequences using UDI indexes, following the protocol and recommendations from the Illumina Truseq kit. The libraries' integrity and quality were checked on TapeStation, screentape D5000, and assayed by qPCR. An equimolar pool of the libraries was then built up using the TruSeq Stranded mRNA Sample Preparation kit from Illumina. Pair-end sequencing (2 × 150 nt) was performed on an Illumina HiSeq 4000 platform at Genewiz.

Clean RNA-seq reads, which had been filtered by Trimmomatic v0.39 (Bolger *et al*., 2014) , were mapped onto the AFL genome assembly using MapSplice (Wang *et al.* 2010) with default parameters. Then the bam files were used with trinity (Grabherr *et al.* 2011) with the option for

genome-guided transcriptome assembly. The obtained SAM alignment files were then converted into binary .BAM files using the samtools suite (Li *et al.* 2009). The quality of the reference transcriptome was then evaluated with Busco (Simão *et al.* 2015).

Structural and functional annotation of the AFL SRA 1002 Genome assembly

Automatic gene prediction was performed using the EuGene Eukaryotic Pipeline (EGNEP v.1.5 with EuGene v.4.2a) and Augustus (Stanke and Morgenstern 2005). EuGene is an integrative gene finder software that is able to combine several sources of information to predict genes (Sallet, Gouzy and Schiex 2019). A first run of Eugene (version 4.2a), was used to mask the genome via the repeat element detection tools integrated in the pipeline. Data from RepBase were provided to Eugene for integration into its genome-wide repeat detection and masking pipeline. The masked sequence was then used for ab-initio prediction via Augustus, with the *A. thaliana* plant model, for the prediction of genes and their structures. The results, in the form of a GFF3 file were incorporated as component in a second run of Eugene via the AnnotaStruct plug-in to generate the finale structural annotation. In addition, transcriptomic and protein evidences were retrieved and provided as input to Eugene. For transcriptomic data we included the AFL SRA 1002 transcriptome assembled by Trinity (of which we kept the longest representative for each transcript) and sequences from different species of the genus Citrus for which we defined very stringent parameters regarding the percentage of coverage (80%) and identity (90%). At the protein level, sequences extracted from the Uniprot database of the taxon Viridiplantae were used. The functional annotation of AFL SRA 1002 genome was performed by assigning their associated generic GO terms with the Blast2GO program using BLAST results from UniProt (UniProt 2021) with E-value 1e-5.

Intragenomic duplication analysis

Intragenomic collinearity analysis was performed using the SynMap pipeline (Haug-Baltzell *et al.* 2017) in the CoGe platform (https://genomevolution.org) to detect segmental duplications in the AFL SRA 1002 genome. Gene pairs were identified using DAGChainer with a maximum distance of 40 genes between matches (-D 40) and a minimum of five colinear gene pairs per block (-A 5). Syntenic blocks were subsequently merged using a maximum gap of 1,000 genes between blocks (-Dm 1000). The resulting duplication data were filtered to retain blocks longer than 7.6 kb (95th percentile) for visualization and downstream analysis. Coordinates and gene content of duplicated segments were extracted for functional assessment.

Annotation of PRR

Protein sequences and corresponding GFF3 annotation files from the AFL genome were used as input data for downstream analyses. Domain annotation of all predicted proteins was performed using InterProScan v5.48-83.0 (Jones *et al.* 2014) with default parameters. The search integrated multiple domain databases, including COILS, Gene3D, HAMAP, MOBIDB, PANTHER, Pfam, PIRSF, PRINTS, ProDom, PROSITE, SFLD, SMART, SUPERFAMILY, and TIGRFAM. Results were exported in tab-separated values (TSV) format.

The identification and classification of pattern recognition receptors (PRRs) and resistance (R) proteins were carried out using RRGPredictor (Silva and Micheli 2020), which assigns each protein to one of 13 structural classes: T, TN, TNL, RPW8NL, RLP, RLK, RLKGNK2, N, NL, CN, CNL, MLO, and UNKNOWN. The UNKNOWN class was used solely for estimating the total number of PRR genes and was excluded from class-specific analyses. Chromosomal localization of genes was visualized using TBtools.

Gene duplication analysis was conducted with MCScanX (Wang *et al.* 2012) under default parameters. Duplication events were analyzed and visualized for each identified PRR and R

gene class, generating both tabular outputs and graphical representations of gene collinearity and tandem duplication.

Annotation of repeated sequences in the genome assembly

The complete diversity of repeated sequences was characterized including the annotation of transposable elements (TEs), satellite DNAs (or tandem repeats), endogenous caulimovirids elements (ECVs) and Simple Sequence Repeats (SSRs). Repeated sequences were identified using the faster method developed by Giraud *et al.* (2025). First, the repeat-finding programs REPET (Quesneville *et al.* 2005; Flutre *et al.* 2011), MITE-Hunter (Han and Wessler 2010) and TAREAN (Novák *et al.* 2017) were used to construct a unique library of consensus sequences consisting of all TEs, ECVs and satellite DNAs identified de novo in the genome assembly. The current library was then compared with the citrus reference repeated sequence library, which was previously developed following the annotation of repeated sequences in 12 *Citrus* species (Giraud 2025). Using BLASTn, all consensus from the current library with a percentage of identity lower than 80% with the consensus of the citrus reference library were retained because they represented new diversity not already found in the reference library (<80% of identity on <80% of the smallest sequences, e-value <1e-06 (Camacho *et al.* 2009). This set of consensus was classified using PASTEC (Hoede *et al.* 2014) and sequences features were checked according to the method developed by (Giraud *et al.* 2025). Finally, the quantity of repeated sequences on the genome assembly and their localization were determined by aligning the citrus reference library enriched with the new consensus set on the pseudoChromosomes using TEannot, a pipeline included in REPET package. The density of the different repeated sequences along pseudoChr omosomes was calculated with a 1-Mb sliding window and represented on circular plot using circos v0.69.9 (Krzywinski *et al.* 2009) (Krzywinski *et al.* 2009).

Supplementary Note 2 : Identification of admixture along the four ancestral genomes

Identification of ancestral species' diagnostic SNPs

We applied the two-step method described in (Ahmed *et al.* 2019) to identify diallelic DSNPs for each of the ten Oceanian and seven Asian main clades as well as the six species associated in three of the main clades. The identification of DSNPs was based respectively on 48 and 55 Oceanian and Asian accessions (Supplementary Table 6).

Previous studies (Oueslati *et al.*, 2017; Wu *et al.*, 2018; Ahmed *et al.*, 2019) have shown that some varieties of the modern horticultural groups were not pure representative of theses ancestral species, but have interspecific introgressions in heterozygosity and even sometime in homozygosity. The first step of the dSNP search was intended to identify introgressions on the representative cultivars. It relied on the heterozygosity level and each accession's similarity to the centroid of each ancestral taxon along the genome. SNPs with a differentiation parameter (GST) value >= 0.5 were selected to contrast areas with and without interspecific introgressions. An example is given in the Figure 1 for five accessions of mandarins on chromosome 2. For Ponkan mandarin, coordinated variations are observed at the end of the chromosome suggesting alternate introgression of *C. maxima* in heterozygosity and homozygosity. Owari mandarin display a large C. maxima introgression in heterozygosity in the middle of Chr2. King mandarin and in a less extent Szibat mandarin display large introgression in heterozygosity at the end of Chr2. No evidence of introgression is found for Cleopatra mandarin and its whole genotyping data were used for DSNPs search. Once the interspecific introgressions are removed (considered as missing data) for each representative accessions, the allelic frequencies in the different ancestral taxa and the GST parameter between each ancestral taxon and the others are

estimated again to select DSNPs with GST = 1 for the differentiation of one of the ancestral species from tall other ones.

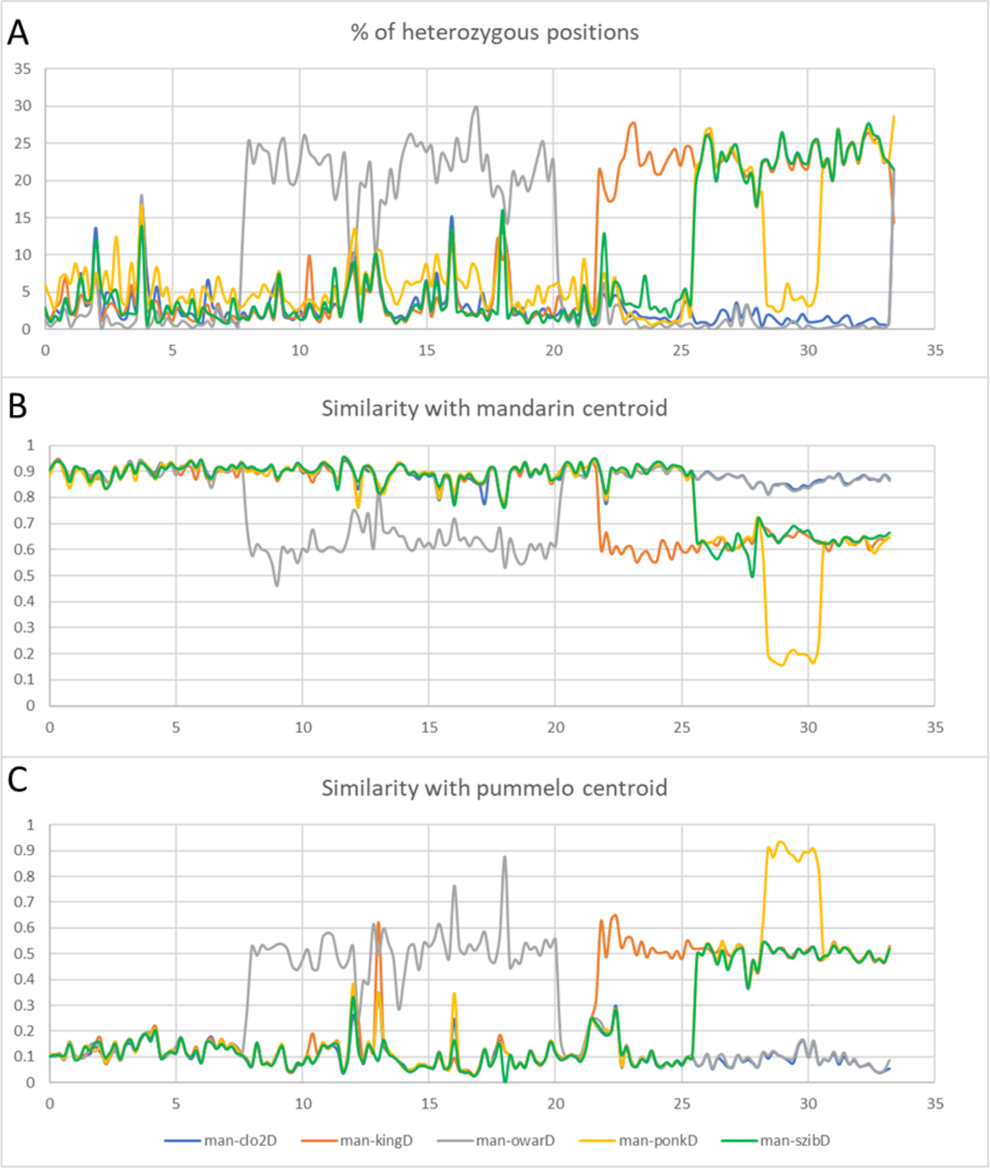

Figure 1: Illustration of the identification of introgressed regions in the chromosome 2 of Microcitrus reference genome for five accessions representative of *C. reticulata* (Cleopatra, King, Owari, Ponkan and Szibat) using sliding windows of 200kb. A: % of heterozygosity for diallelic SNP with GST value > 0.5 for one of the ancestral species; B: similarity with the centroid of *C. reticulata* estimated from diallelic SNPs with GST value > 0.5 for *C. reticulata*; C: similarity with the centroid of *C. maxima* estimated from diallelic SNPs with GST value > 0.5 for *C. reticulata* or *C. maxima*.

Admixture along the four ancestral genomes

TraceAncestor software (Ahmed et al., 2019) was adapted for WGS data from diploid genotypes. Genotyping data were used instead of read counts to conduct maximum-likelihood

tests of each ancestor contribution (i.e., allelic doses = 0, 0.5, 1 dose) in genomic windows of 20 DSNPs.

Considering A as the ancestral taxon allele at each locus, B as the alternative allele (allele from another ancestor), and e as the sequencing error rate (fixed at 0.01 for our analysis), the probability of observing i ancestral alleles and j alternative allele for the 20 DSNP loci according to the tested model of ancestral doses for diploid plants was as follows:

$$\text{2 ancestral doses (AA): } C \times P(A)^i \times P(B)^j = C \times (1-e)^i \times e^j$$

$$\text{1 ancestral dose (AB): } C \times P(A)^i \times P(B)^j = C \times 0.5^i \times 0.5^j$$

$$\text{0 ancestral dose (BB): } C \times P(A)^i \times P(B)^j = C \times e^i \times (1-e)^j$$

where C is a combinatorial coefficient which depends on the observed data but remains constant between the models; P(A) and P(B) respectively the theoretical frequencies of the ancestor and alternative alleles.

We compared one dose hypothesis (heterozygosity) with 0 and two dose hypotheses by calculating a LOD (logarithm of odds) score :

$$\text{LOD } (0/1) = \text{Log10 } ((e^i \times (1-e)^j)/(0.5^i \times 0.5^j))$$

$$\text{LOD } (1/2) = \text{Log10 } ((0.5^i \times 0.5^j)/((1-e)^i \times e^j))$$

t being the selected LOD threshold value, the process to conclude for a dose was as follows:

If LOD (0/1) >= t : 0 ancestral taxon dose

If – t < LOD < t : undetermined dose

If LOD (0/1) <= - t and LOD (1/2) >= t : 1 ancestral taxon dose

If LOD (0/1) <= - t and –t < LOD (1/2) < t : undetermined dose

If LOD (0/1) <= - t and LOD (1/2) <= - t : 2 ancestral taxa doses

The t value (LOD threshold) was fixed at 3 for all analysis

During this step, the number and position of windows varied between the ancestral species according to the density and positions of the DSNPs of each of the considered species. To integrate the information obtained for all ancestral species, we sub-divided the genome into successive fragments of 100 kb. For each ancestor and for each 100 kb genomic fragment, the corresponding window of 20 DSNPs was identified and the ancestral dose of this window was attributed to the considered 100 kb genomic fragment. In cases in which the sum of ancestral contributions exceeded 2, the corresponding windows were considered undetermined for the two haplotypes. The identification of DSNPs and mosaic structure analyses were performed using the SniPlay web tool (https://sniplay.southgreen.fr/cgi-bin/home.cgi).

Bibliography

Ahmed D et al. Genotyping by sequencing can reveal the complex mosaic genomes in gene pools resulting from reticulate evolution: a case study in diploid and polyploid citrus. *Ann Bot* 2019;123:1231–1251.

Aury J-M, Istace B. Hapo-G: haplotype-aware polishing of genome assemblies with accurate reads. *NAR Genom Bioinform* 2021;3:lqab034.

Baurens F-C et al. Recombination and large structural variations shape interspecific edible banana genomes. *Mol Biol Evol* 2019;36:97–111.

Bolger AM et al. Trimmomatic: a flexible trimmer for Illumina sequence data. *Bioinformatics* 2014;30:2114–2120.

Camacho C et al. BLAST+: architecture and applications. *BMC Bioinformatics* 2009;10:421.

Chen Y et al. Efficient assembly of nanopore reads via highly accurate and intact error correction. *Nat Commun* 2021;12:60.

Elshire RJ et al. A robust, simple genotyping-by-sequencing (GBS) approach for high diversity species. *PLoS ONE* 2011;6:e19379.

Flutre T et al. Considering transposable element diversification in de novo annotation approaches. *PLoS ONE* 2011;6:e16526.

Giraud D. Library of repeated sequences annotated in Citrus genome assemblies. 2025. https://doi.org/10.57745/ZVYEBU

Giraud D et al. Construction of a comprehensive library of repeated sequences for the annotation of Citrus genomes. *BMC Genom Data* 2025;26:30.

Grabherr MG et al. Full-length transcriptome assembly from RNA-Seq data without a reference genome. *Nat Biotechnol* 2011;29:644–652.

Han Y, Wessler SR. MITE-Hunter: a program for discovering miniature inverted-repeat transposable elements from genomic sequences. *Nucleic Acids Res* 2010;38:e199.

Haug-Baltzell A et al. SynMap2 and SynMap3D: web-based whole-genome synteny browsers. *Bioinformatics* 2017;33:2197–2198.

Hoede C et al. PASTEC: an automatic transposable element classification tool. *PLoS ONE* 2014;9:e91929.

Istace B et al. BiSCoT: improving large eukaryotic genome assemblies with optical maps. *PeerJ* 2020;8:e10150.

Jones P et al. InterProScan 5: genome-scale protein function classification. *Bioinformatics* 2014.

Krzywinski M et al. Circos: an information aesthetic for comparative genomics. *Genome Res* 2009;19:1639–1645.

Li H et al. The sequence alignment/map format and SAMtools. *Bioinformatics* 2009;25:2078–2079.

Luro F et al. The INRA-CIRAD citrus germplasm collection of San Giuliano, Corsica. In: Zech-Matterne V, Fiorentino G (eds.), *AGRUMED: Archaeology and History of Citrus Fruit in the Mediterranean*. Naples: Publications du Centre Jean Bérard; 2017.

Novák P et al. TAREAN: a computational tool for identification and characterization of satellite DNA from unassembled short reads. *Nucleic Acids Res* 2017;45:e111.

Quesneville H et al. Combined evidence annotation of transposable elements in genome sequences. *PLoS Comput Biol* 2005;1:e22.

Sallet E et al. EuGene: an automated integrative gene finder for eukaryotes and prokaryotes. In: Kollmar M (ed.), *Gene Prediction*. New York: Springer; 2019. p. 97–120.

Silva RJS, Micheli F. RRGPredictor, a set-theory-based tool for predicting pathogen-associated molecular pattern receptors (PRRs) and resistance (R) proteins from plants. *Genomics* 2020;112:2666–2676.

Simão FA et al. BUSCO: assessing genome assembly and annotation completeness with single-copy orthologs. *Bioinformatics* 2015;31:3210–3212.

Stanke M, Morgenstern B. AUGUSTUS: a web server for gene prediction in eukaryotes that allows user-defined constraints. *Nucleic Acids Res* 2005;33:W465–W467.

Wang K et al. MapSplice: accurate mapping of RNA-seq reads for splice junction discovery. *Nucleic Acids Res* 2010;38:e178.

Wang Y et al. MCScanX: a toolkit for detection and evolutionary analysis of gene synteny and collinearity. *Nucleic Acids Res* 2012;40:1–14
