# Supplementary material for: Chromosome-scale genome assembly of Australian finger lime and resequencing reveal the hidden diversity of Oceanian citrus
Source: Hortic Res. 2026 Jun 2;13(11):uhag213. doi: 10.1093/hr/uhag213 (PMC13719177; doi:10.1093/hr/uhag213)
Supplement: Supplementary file 1 [file web_material_uhag213.zip › Supplementary Tables_ Hufnagel_etal_fev26.pdf]

Supplementary Table 1. Summary statistics of the Australian finger lime (SRA 1002) genome scaffold assembly.

Statistic	Value (Mbp or %)
Total number of scaffolds	403
Cumulative scaffold size	450.8 Mbp
N50 scaffold size	28.40 Mbp
Number of scaffolds ≥ N50	7
N80 scaffold size	5.41 Mbp
Number of scaffolds ≥ N80	19
N90 scaffold size	0.72 Mbp
Number of scaffolds ≥ N90	47
Minimum scaffold size	0.011 Mbp
Maximum scaffold size	38.55 Mbp
Average scaffold size	1.12 Mbp
auN (area under the Nx curve)	21.14 Mbp
% GC content	34.72%
% N bases	6.48%

Supplementary Table 2: *C. australis* × *C. inodora* genetic map: distribution of the SNP markers among linkage groups and AFL pseudochromosomes

LG\SC	1	2	3	4	5	6	7	8	9	Mks/LG	LG size cM
1	266	0	0	0	0	0	0	0	0	266	106.28
2	0	313	0	0	0	0	0	0	0	313	123.47
3	0	0	469	0	0	0	0	0	0	469	155.44
4	0	0	0	338	0	0	0	0	0	338	103.82
5	0	1	0	0	302	0	1	0	2	306	94.03
6	0	0	1	0	0	241	0	0	0	242	77.36
7	1	0	0	2	0	0	256	0	0	259	92.1
8	0	0	0	0	0	0	0	230	0	230	95.81
9	0	0	1	0	0	0	0	0	198	199	95.3
Mks /Sc	267	314	471	340	302	241	257	230	200	2,622	943.61

Supplementary Table 3 : Results of the *AFL SRA 1002* reference transcriptome implementation

Sample	Sequencing Volume (Gb)	Nb of transcripts	BUSCO score
Flowers	67.8	76 534	95
Fruit pulp	23	43 806	76
Mature leaves	51.6	58 892	91
Young leaves	54	54 718	94

Supplementary Table 4: Classification of new repeated sequences identified on the AFL SRA 1002 genome assembly

		Number of consensus sequences	Number of copies (full / truncated)	Genome coverage (%)
**Retrotransposons (Class I elements)**				
LTR *Copia* (RLC)	*Tork*	4	4 / 191	0.051%
	Retrofit	3	17 /17	0.037%
LTR *Gypsy* (RLG)	*Tat*	1	0 / 6	0.000%
	Crm	1	0 / 9	0.001%
LINEs (RIX)		6	9 / 902	0.067%
**DNA transposons (Class II elements)**				
TIRs	*Mutator* (DTM)	2	4 / 36	0.011%
	Pif-Harbinger (DTH)	1	0 / 27	0.002%
	Tc1-Mariner (DTT)	1	2 / 25	0.003%
Unclassified MITEs (DXX-MITE)		5	8 /11	0.000%
Helitron (DHX)		1	4 / 4	0.001%
**TOTAL Transposable Elements**		**25**	**48 / 1128**	**0.173%**

Supplementary Table 5: Comparative overview of immune receptor repertoires across citrus genomes.

Panel A – PRR repertoire identified using the RRGPredictor pipeline

Genome / Species	CN	CNL	MLO	N	NL	RLK	RLKGNK2	RLP	RPW8NL	T	TN	TNL	UNKNOWN	TOTAL PRR
**AFL SRA1002**	25	135	17	27	74	54	21	12	4	29	13	52	816	**1284**
***C. reticulata* (CITRE) ***	37	250	17	44	147	102	30	26	12	19	11	56	1263	**2014**
C. maxima* (CITMA)	41	227	21	48	104	95	32	18	7	14	5	19	1220	**1851**
C. micrantha* (CITMI)	20	182	17	52	100	97	35	17	6	35	26	73	1239	**1899**
C. medica* (CITME)	37	139	18	54	83	88	36	25	14	33	15	55	1030	**1627**

PRR genes were identified using InterProScan v5.48–83.0 and classified using RRGPredictor into 13 classes following Santana Silva & Micheli (2020). * Data from Droc et al. 2026

Panel B – NLR genes identified by NB-ARC domain search (Singh et al. 2024)

Species	CNL	TNL	NL	Total NLR
***C. australasica* (haplotype a)ǂ**	195	119	262	**576**
***C. australasica* (haplotype b) ǂ**	172	129	244	**545**
***C. glauca* (haplotype a) ǂ**	114	105	230	**449**
***C. glauca* (haplotype b) ǂ**	116	112	231	**459**
***C. inodora* (haplotype a) ǂ**	109	69	141	**319**
***C. inodora* (haplotype b) ǂ**	107	71	147	**325**
***C. clementina*ǂ**	205	104	270	**579**
***C. sinensis*ǂ**	214	145	292	**651**

NLR genes were identified by detecting the NB-ARC domain (PF00931) using Pfam and confirmed with InterPro domain annotation. ǂ Data from Singh et al. 2024

Supplementary Table 6. Number of duplication events of PRR receptors and R genes present in each class in AFl SRA 899 genome assembly. Genes involved in two duplication events are highlighted in bold.

Class	Quantity of duplication events	Duplication events
CN	1	Ciaus5g05200:Ciaus5g05670
CNL	4	Ciaus5g14600:**Ciaus5g18130**<br>Ciaus5g17860:**Ciaus5g18130**<br>Ciaus5g16120:**Ciaus5g16660**<br>Ciaus5g16040:**Ciaus5g16660**
MLO	1	Ciaus5g40430:Ciaus7g00190
N	0	-
NL	4	Ciaus5g01860:Ciaus5g02490<br>Ciaus5g02180:Ciaus5g02790<br>Ciaus5g14630:Ciaus5g17990<br>Ciaus5g05320:Ciaus5g05760
RLK	1	Ciaus6g16950:Ciaus6g17410
RLKGNK2	2	Ciaus2g08190:**Ciaus4g25280**<br>Ciaus3g05430:**Ciaus4g25280**
RLP	0	-
RPW8NL	0	-
T	2	Ciaus2g02190:Ciaus2g02750<br>Ciaus5g08260:Ciaus5g08640
TN	0	-
TNL	3	Ciaus3g34420:Ciaus3g35380<br>Ciaus3g30790:Ciaus3g31190<br>Ciaus5g08170:Ciaus5g08580

Supplementary Table 8: DSNPs of the species of the *C. wakonai* + *C. warburgiana C. garrawayi* + Unknown and *C. medica* + *C. indica* main clades

	**Chr1**	**Chr2**	**Chr3**	**Chr4**	**Chr5**	**Chr6**	**Chr7**	**Chr8**	**Chr9**	**Total**
C. wakonai	10535	11471	13261	9074	8202	8396	9157	7992	6938	**85026**
C. warburgiana	11079	16095	14531	7831	6405	9875	10307	8429	9255	**93807**
Unknown	12081	12407	16665	8244	9684	13664	8468	11747	7095	**100055**
C. garrawayi	7082	8952	10753	5168	6803	8731	5364	8184	4383	**65420**
C. medica	27681	28322	38403	27610	23379	25537	21649	24197	22345	**239123**
C. indica	15287	16623	22038	15205	12822	13589	12840	13619	11760	**133783**
Total	83745	93870	115651	73132	67295	79792	67785	74168	61776	**717214**

Supplementary Table 9: Manual functional categorization of gene families

Functional category	***Biological interpretation***	***Annotation basis / representative keywords***
Disease-resistance modules	Canonical plant immune receptors and resistance-related proteins involved in pathogen recognition and activation of defense responses	NB-LRR, NBS-LRR, TNL, CNL, resistance protein, R gene
Regulatory proteins (TF/PPR)	Transcriptional and post-transcriptional regulators controlling gene expression	transcription factor, TF, pentatricopeptide repeat, PPR
Protein kinases	Intracellular signaling components involved in phosphorylation cascades	kinase, serine/threonine kinase, protein kinase
Cytochrome P450 / specialized metabolism	Enzymes involved in secondary metabolism, detoxification, and biosynthetic diversification	cytochrome P450, monooxygenase, oxidoreductase

Functional categories were manually curated to summarize enriched gene families shown in Fig. 6B. Gene families were assigned to categories based on annotation outputs.
